# Perspectives of Hispanic and Latinx Community Members on AI-Enabled mHealth Tools: Qualitative Focus Group Study

**DOI:** 10.2196/59817

**Published:** 2025-02-06

**Authors:** Stephanie A Kraft, Shaan Chopra, Miriana C Duran, Janet A Rojina, Abril Beretta, Katherine I López, Russell Javan, Benjamin S Wilfond, Margaret Rosenfeld, James Fogarty, Linda K Ko

**Affiliations:** 1 Department of Bioethics and Decision Sciences Geisinger College of Health Sciences Danville, PA United States; 2 Paul G. Allen School of Computer Science & Engineering University of Washington Seattle, WA United States; 3 Department of Health Systems and Population Health University of Washington School of Public Health Seattle, WA United States; 4 Treuman Katz Center for Pediatric Bioethics and Palliative Care Seattle Children's Research Institute Seattle, WA United States; 5 Department of Pediatrics University of Washington School of Medicine Seattle, WA United States; 6 Center for Respiratory Biology and Therapeutics Seattle Children’s Research Institute Seattle, WA United States

**Keywords:** wearable electronic devices, qualitative research, mobile health, mHealth, digital health, privacy, data sharing, artificial intelligence, AI, community, chronic conditions, chronic disease

## Abstract

**Background:**

Mobile health (mHealth) tools have the potential to reduce the burden of chronic conditions that disproportionately affect Hispanic and Latinx communities; however, digital divides in the access to and use of health technology suggest that mHealth has the potential to exacerbate, rather than reduce, these disparities.

**Objective:**

A key step toward developing health technology that is accessible and usable is to understand community member perspectives and needs so that technology is culturally relevant and appropriately contextualized. In this study, we aimed to examine the perspectives of Hispanic and Latinx community members in Washington State about mHealth.

**Methods:**

We recruited English- and Spanish-speaking Hispanic or Latinx adults to participate in web-based focus groups through existing community-based networks across rural and urban regions of Washington State. Focus groups included a presentation of narrative slideshow materials developed by the research team depicting mHealth use case examples of asthma in children and fall risk in older adults. Focus group questions asked participants to respond to the case examples and to further explore mHealth use preferences, benefits, barriers, and concerns. Focus group recordings were professionally transcribed, and Spanish transcripts were translated into English. We developed a qualitative codebook using deductive and inductive methods and then coded deidentified transcripts using the constant comparison method. The analysis team proposed themes based on review of coded data, which were validated through member checking with a community advisory board serving Latino individuals in the region and finalized through discussion with the entire research team.

**Results:**

Between May and September 2023, we conducted 8 focus groups in English or Spanish with 48 participants. Focus groups were stratified by language and region and included the following: 3 (n=18, 38% participants) Spanish urban groups, 2 (n=14, 29% participants) Spanish rural groups, 1 (n=6, 13% participants) English urban group, and 2 (n=10, 21% participants) English rural groups. We identified the following seven themes: (1) mHealth is seen as beneficial for promoting health and peace of mind; (2) some are unaware of, unfamiliar with, or uncomfortable with technology and may benefit from individualized support; (3) financial barriers limit access to mHealth; (4) practical considerations create barriers to using mHealth in daily life; (5) mHealth raises concern for overreliance on technology; (6) automated mHealth features are perceived as valuable but fallible, requiring human input to ensure accuracy; and (7) data sharing is seen as valuable for limited uses but raises privacy concerns. These themes illustrate key barriers to the benefits of mHealth that communities may face, provide insights into the role of mHealth within families, and examine the appropriate balance of data sharing and privacy protections.

**Conclusions:**

These findings offer important insights that can help advance the development of mHealth that responds to community values and priorities.

## Introduction

### Background

Mobile health (mHealth) tools, broadly defined to include patient-facing, health-related mobile or wireless devices, such as sensors, trackers, and wearables [1,2], offer the potential to improve health care access and encourage health-promoting behaviors through personal health monitoring. In particular, mHealth powered by artificial intelligence to make actionable, personalized predictions is a growing field that aims to advance broad-reaching, patient-driven preventive care [3]. For example, recent work has demonstrated the efficacy of an artificial intelligence–aided home stethoscope in detecting asthma exacerbations in children, which can facilitate time-sensitive decisions about management [4]. Others are using mHealth monitoring devices, such as smart inhalers and smartwatches, to collect personal and environmental data to facilitate the development of predictive models for asthma attacks [5,6]. Mobile data collection and device availability beyond clinical settings may be particularly valuable for overcoming geographic, financial, and other structural barriers to health care that disproportionately affect marginalized and minoritized groups, including Hispanic and Latinx communities [7].

mHealth tools have the potential to reduce disease burden and have been shown to be especially well suited for managing chronic health conditions, such as asthma, and particularly pediatric asthma [8-11], by facilitating continuous monitoring [12]. In the United States, pediatric asthma disproportionately affects Hispanic and Latinx children. An analysis of health record data from community health centers across 18 states showed Spanish-preferring Latinx children have a higher likelihood of clinic visits for asthma exacerbations [13], and overall, Hispanic children have a 40% higher death rate from asthma compared to non-Hispanic White children [14]. Existing mHealth tools for pediatric asthma monitor a wide range of factors, including air quality, lung function, physical activity, sleep, and cough [15]. Hispanic individuals in the United States own smartphones at similar rates to other racial and ethnic groups and are more likely than White individuals to use a smartphone as their primary means of internet access [16]. However, at least 1 recent cross-sectional study has shown that Hispanic and Latinx families are significantly less likely to have reliable, high-speed internet as compared to White families [17], illustrating the persistence of the digital divide that imposes disproportionate barriers to accessing digital technologies such as mHealth. For example, an evaluation of remote digital studies found that minoritized groups, including Hispanic and Latinx individuals, were overrepresented in clusters with lower app use, suggesting a need for improved engagement approaches [18]. The digital divide may be even greater among those living in rural areas [16]. These patterns suggest that digital technologies may replicate and exacerbate existing disparities if devices are not implemented in a manner that is culturally appropriate and responsive to individuals’ social determinants and lived realities [19-22]. Cultural appropriateness is essential to ensure a tool is acceptable and effective for its target population and can work toward maximizing meaningful benefits and minimizing important risks [23,24]. Likewise, sensitivity to how social and economic structures differentially shape populations’ access to and use of health technology is necessary to support fairness when implementing devices into real-world settings [24]. A 2018 systematic literature review found that SMS text message–based mHealth tools were consistently studied among marginalized populations; however, there were gaps in studies of other mHealth strategies as well as their real-world implementation [25].

### This Study

As a step toward informing health technology that is responsive to individuals’ sociocultural contexts, we conducted an in-depth focus group study to provide formative research on the values and lived experiences of Hispanic and Latinx community members in Washington State as they relate to mHealth. We used focus group methods, incorporating relevant mHealth case examples, to allow for group discussion and deliberation [26]. Our specific aim was to describe the perspectives of Hispanic and Latinx community members in rural and urban regions of Washington State about mHealth.

## Methods

### Participants

We recruited participants through community-based networks in urban western Washington and rural central Washington in several ways. First, we contacted people who had signed up to be contacted about research opportunities on the Community Voices registry, a longstanding partnership with Hispanic communities in central Washington led by a study team member (LKK). Second, we contacted previous participants in other studies who had agreed to be contacted for future research. Third, we shared recruitment flyers with networks serving local Hispanic and Latinx communities.

Individuals were eligible to participate if they were aged ≥18 years, spoke either English or Spanish, and identified as Hispanic, Latino, Latina, or Latinx. All study materials were offered in both English and Spanish, and bilingual study team members were available for all participant interactions [27]. Participants completed a brief demographic questionnaire.

### Focus Groups

We developed a focus group guide (Multimedia Appendix 1) to explore key topics related to health technology use, including potential patient-level barriers and concerns as well as relevant mHealth design decisions, drawing on our multidisciplinary team’s expertise in these topics. Our approach was also informed, in part, by the lived informatics model, which delineates a process through which people decide to use, experience using, and may ultimately abandon technology in real-world settings [28].

To ensure all participants had a shared understanding to foster robust deliberation [29], we created multimedia informational aids to present during the focus groups. We collaborated with Booster Shot Media, a private health-focused communication company comprised of a health communication expert and a pediatrician who is also a cartoonist with over a decade of experience developing multimedia tools for use in patient-centered research, to develop a series of illustrated narrative slideshows depicting Latinx families using mHealth devices. Our team first identified several clinical scenarios that would reflect realistic near-future uses of mHealth and would also be relevant to focus group participants’ lived experiences. These scenarios were based on our collective knowledge of the communities where we planned to recruit and of mHealth development. Through iterative team discussion, we identified pediatric asthma as a paradigmatic example of a condition that disparately impacts Hispanic and Latinx populations in the United States, which is common in the region where we conducted our study and has several distinct opportunities for mHealth intervention. We also identified a second example case of fall risk in older adults as similarly amenable to mHealth intervention and relevant to this population. While we used these 2 example cases to provide tangible, relevant starting points for discussion, conversations were not limited to these clinical cases; instead, our discussion questions built on and expanded from these cases.

**Figure 1:**
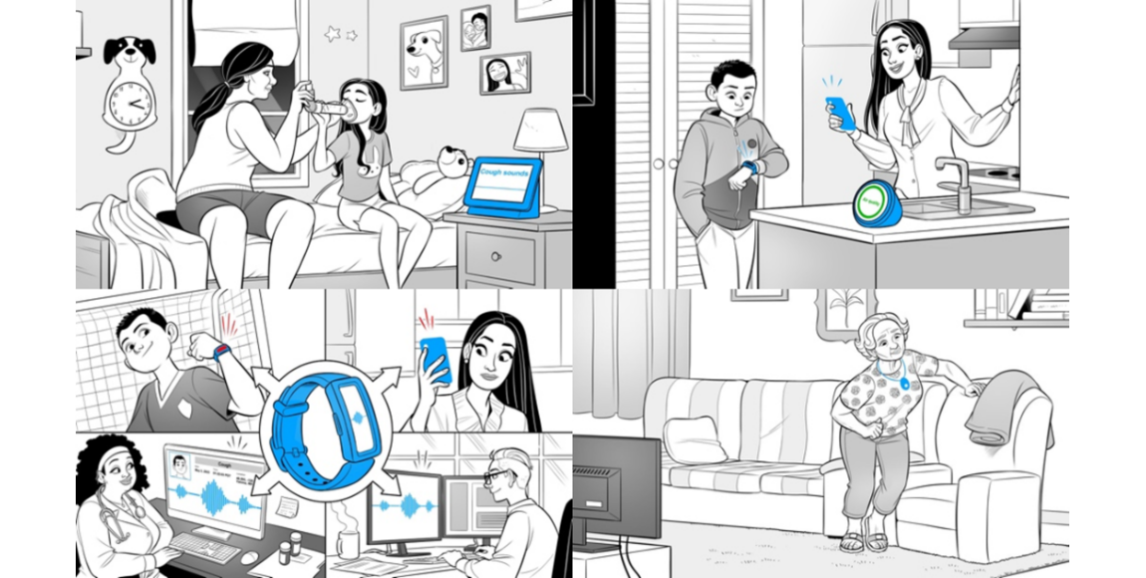
Example narrative slideshow of families using mobile health to control pediatric asthma and monitor fall risk in an older adult. The slideshows were developed in collaboration with Booster Shot Media.

We developed the narratives of our slideshows by drawing on team members’ personal and professional experiences with Latinx families as well as professional experiences with mHealth and pediatric asthma, and we worked with Booster Shot Media to finalize and illustrate the narratives. Booster Shot Media reviewed photos of Latinx individuals living in the regions where we recruited participants to create culturally relevant illustrations, which were refined through iterative review and feedback from team members with direct experience with the communities where we recruited. We also sought feedback on the slideshows from bilingual and bicultural colleagues outside of our study team. The final versions of the slideshows first presented concrete examples of how mHealth could be used to predict asthma exacerbations and manage asthma in children, how different devices might work, and how data might be shared. They described these examples through a family-based narrative showing parents and children working together to manage pediatric asthma. The second example showed an older adult, the family’s “abuelita” (grandmother), using a wearable device to predict fall risk, which was a briefer narrative intended to explore potential differences in perspectives based on the individual using the device and the condition being tracked. These examples described how the depicted mHealth tools could make personalized predictions and improve those predictions based on collected data, but we did not explicitly use the term “artificial intelligence” to avoid raising potentially preconceived notions about artificial intelligence as depicted in the broader media. Example slideshow images are shown in Figure 1. The full slide decks and text are included in Multimedia Appendix 2 (English version) and Multimedia Appendix 3 (Spanish version).

Focus groups were held on Zoom (Zoom Video Communications) and moderated by at least 2 members of the research team, with at least 1 additional team member present for note-taking and technology support. Native speakers in the research team facilitated the Spanish focus groups. Audio recordings were professionally transcribed verbatim, and Spanish focus groups were professionally translated into English. Transcripts were reviewed by the research team for accuracy of transcription and translation, deidentified, and uploaded to the cloud-based platform Dedoose (SocioCultural Research Consultants, LLC) [30] for qualitative analysis management.

We developed an initial codebook based on a deductive review of the focus group moderator guide topic areas. Two team members (MCD and SC) reviewed 2 focus group transcripts, revised the initial codebook using inductively derived codes from the transcripts, and coded those transcripts using the constant comparison method [31]. They continued this iterative process with additional transcripts until the codebook needed no further revision. A third coder (AB) was then trained. All transcripts were coded by the coding team and reviewed for consensus.

Coded excerpts were extracted for review and identification of themes [32,33]. We divided excerpts for review and summarization following the codebook structure, as our initial review suggested that themes aligned with the previously identified codes. This step allowed us to identify key themes and condense the data to facilitate reporting. Two team members (MCD and SC) summarized a portion of the excerpts and proposed themes, and to ensure credibility, a third member (SAK) independently summarized and proposed themes from all excerpts. Proposed themes were compared and discussed among the analysis team, which included a community-based researcher, a computer scientist, and a bioethicist. Our coding team additionally included a bioethics research assistant, and 2 of 3 coders were bilingual in English and Spanish. A summary of themes was shared with a community advisory board serving Latino individuals in central Washington for member checking, which validated and identified important nuances in our findings [34]. We then revised and finalized our themes through discussion with the entire research team.

### Ethical Considerations

This study was approved as exempt by the institutional review boards at the University of Washington (STUDY00016183) and Seattle Children’s Hospital (STUDY00004107). All participants reviewed an information sheet with a study team member in their preferred language and provided verbal consent. All transcripts were deidentified before analysis, and only deidentified quotes were reported. Participants received a US $50 gift card following the focus group.

## Results

### Participants

We conducted 8 focus groups: 5 in Spanish, 3 in English, and 4 each with rural and urban participants. These focus groups included 48 participants overall, with an average of 6 (SD 2) participants per focus group and a range of 3 to 9. Participants reported a mean age of 45 (SD 14) years, and most (43/48, 90%) were women. Most (38/48, 79%) indicated they were born outside the United States, including nearly all (31/32, 97%) participants in the Spanish focus groups and half (5/10, 50%) of participants in the rural English focus groups. Most (29/48, 60%) participants reported having either no health insurance or state-funded health insurance, and a majority (31/48, 65%) reported no formal post–high school education. Detailed participant characteristics, including self-reported experience with mHealth and asthma, are shown in Table 1.

**Table 1:** Participant characteristics (N=48).

		Spanish, urban (n=18)	Spanish, rural (n=14)	English, urban (n=6)	English, rural (n=10)	Total (N=48)
Age (y), mean (SD)		54 (13)	42 (11)	33 (9)	39 (11)	45 (14)
**Gender identity, n (%)**						
	Women	16 (89)	13 (93)	5 (83)	9 (90)	43 (90)
	Men	2 (11)	1 (7)	1 (17)	1 (10)	5 (10)
**Race or ethnicity (check all that apply), n (%)**						
	Native American or Alaska Native	1 (6)	0 (0)	0 (0)	0 (0)	1 (2)
	Hispanic, Latino, or of Spanish origin	17^a^ (94)	14 (100)	6 (100)	10 (100)	47^a^ (98)
	White	1^a^ (6)	0 (0)	0 (0)	0 (0)	1^a^ (2)
**Birthplace, n (%)**						
	Outside the United States^b^	17 (94)	14 (100)	2 (33)	5 (50)	38 (79)
**Educational attainment, n (%)**						
	Elementary school or lower	3 (17)	6 (43)	0 (0)	0 (0)	9 (19)
	Some high school	3 (17)	1 (7)	0 (0)	1 (10)	5 (10)
	High school graduate or GED^c^	9 (50)	5 (36)	1 (17)	2 (20)	17 (35)
	Technical school diploma	1 (6)	0 (0)	0 (0)	0 (0)	1 (2)
	Some college	0 (0)	0 (0)	0 (0)	2 (20)	2 (4)
	College graduate	2 (11)	2 (14)	1 (17)	2 (20)	7 (15)
	Graduate school degree	0 (0)	0 (0)	4 (67)	3 (30)	7 (15)
**Health insurance (check all that apply), n (%)**						
	Employer-sponsored	3 (17)	4 (29)	3 (50)	6 (60)	16 (33)
	Medicare	3^a^ (17)	0 (0)	1 (17)	0 (0)	4^a^ (8)
	Medicaid, Washington Apple Care, or coupons	4^a^ (22)	3 (21)	0 (0)	3 (30)	10^a^ (21)
	None	8 (44)	6 (43)	1 (17)	1 (10)	16 (33)
	Other	1 (6)	0 (0)	1 (17)	0 (0)	2 (4)
	Prefer not to answer	0 (0)	1 (7)	0 (0)	0 (0)	1 (2)
**Annual household income (US $), n (%)**						
	<15,000	1 (6)	1 (7)	0 (0)	0 (0)	2 (4)
	15,000 to <30,000	2 (11)	3 (21)	0 (0)	2 (20)	7 (15)
	30,000 to <45,000	1 (6)	2 (14)	0 (0)	2 (20)	5 (10)
	45,000 to <60,000	3 (17)	0 (0)	0 (0)	1 (10)	4 (8)
	60,000 to <75,000	1 (6)	1 (7)	4 (67)	1 (10)	7 (15)
	≥75,000	0 (0)	2 (14)	2 (33)	4 (40)	8 (17)
	Do not know or prefer not to answer	10 (56)	5 (36)	0 (0)	0 (0)	15 (31)
**Have you or someone you know been diagnosed with asthma? (check all that apply), n (%)**						
	Yes, I have been diagnosed with asthma	3 (17)	2 (14)	0 (0)	2^a^ (20)	7^a^ (15)
	Yes, I have a family member or close friend with asthma	6 (33)	3 (21)	6^a^ (100)	8^a^ (80)	23^a^ (48)
	Yes, I know someone else with asthma	3 (17)	2 (14)	2^a^ (33)	0 (0)	7 (15)
	No	6 (33)	7 (50)	0 (0)	1 (10)	15 (31)
**How comfortable are you with using mobile devices, for example, smartphones? n (%)**						
	Very comfortable	9 (50)	6 (43)	5 (83)	6 (60)	26 (54)
	Somewhat comfortable	5 (28)	6 (43)	1 (17)	3 (30)	15 (31)
	Not very comfortable	2 (11)	1 (7)	0 (0)	1 (10)	4 (8)
	Not at all comfortable	2 (11)	1 (7)	0 (0)	0 (0)	3 (6)
**Have you ever used any technology to track your personal health or wellness, for example, apps or devices related to diet, sleep, exercise, or steps? n (%)**						
	Yes, I currently use technology to track my health or wellness	7 (39)	5 (36)	4 (67)	6 (60)	22 (46)
	Yes, I have used technology to track my health or wellness in the past but do not use it currently	1 (6)	3 (21)	1 (17)	2 (20)	7 (15)
	No, I have never used technology to track my health or wellness	10 (56)	6 (43)	1 (17)	2 (20)	19 (40)

^a^At least 1 participant selected this category and at least 1 additional category on a question with a “Check all that apply” instruction.

^b^Participants (N=48) indicated the following countries of origin: Mexico (n=35, 73%), Argentina (n=2, 4%), and Colombia (n=1, 2%).

^c^GED: General Educational Development test.

### Focus Group Themes

#### Overview

We identified seven overarching themes from our focus groups: (1) mHealth is seen as beneficial for promoting health and peace of mind; (2) some are unaware of, unfamiliar with, or uncomfortable with technology and may benefit from individualized support; (3) financial barriers limit access to mHealth; (4) practical considerations create significant barriers to using mHealth in daily life; (5) mHealth raises concern for overreliance on technology; (6) automated mHealth features are perceived as valuable but fallible, requiring human input to ensure accuracy; and (7) data sharing is seen as valuable for limited uses but raises privacy concerns.

We examined the data for differences in themes based on rural or urban location and English and Spanish language but did not identify distinct themes in any subgroups. Illustrative quotes for each theme are provided throughout.

#### Theme 1: mHealth Is Seen as Beneficial for Promoting Health and Peace of Mind

Most participants described mHealth as beneficial, discussing how this technology could help track chronic conditions and prevent serious health events through earlier intervention. For example, 1 participant noted the potential value of tracking their health conditions:

I would think it would be fantastic because I suffer from an autoimmune disease, I have no balance, so I have suffered several falls, and that would be a help for me, a help to be alert all the time. I would love to have that device.

Urban; Spanish-speaking participant; translated; FG US3

Another described how mHealth would have helped their family navigate interactions with their physician to improve their child’s care:

I insisted a lot with the doctors. Because they all sent [my child with asthma] home, that his cough was normal, that his cough was temporary...I wish someone had given us that monitor, because we would go to the doctor and they would say: “And how is his cough? And how often is he coughing?” And I’d be like, “We’ve already had so many months without being able to sleep, like the least I pay attention to is the cough, what the cough sounds like.” Yes, I would have liked to have had a device so it would have been easier for the doctor to know what his cough was like.

Urban; Spanish-speaking participant; translated; FG US3

One participant described the potential impact of earlier detection on their rural setting:

Here in [rural area], we only have one hospital, the emergency room is packed sometimes. We don’t have enough beds on the floors. I feel it would really benefit in preventing people from going really bad to the hospital and flooding the hospital with people. Instead, they can use their devices and go to their doctor, take a little better care of their health or at least keep track of—especially with chronic issues. They can monitor if they’re starting to notice some changes, then they can act on it earlier than later.

Rural; English-speaking participant; FG RE2

Many participants also discussed that these tools could promote peace of mind and feelings of safety. They linked the presented examples to their own experiences caring for children with asthma or supporting older family members in living independently, highlighting how technology could allow them to feel comfortable without needing to continuously monitor their family members. Several participants reflected on their fears as parents of children with asthma:

I used to take [my children] to my room so I can be watching them because it was scary. And especially at night, because during the day, they were fine. But at night, they usually had those asthma attacks when they were little. And we ended up in the hospital...As they get older, I mean, they tell you or they get out of their bedroom or whatever to tell you. But when they’re little, they don’t. So you have to be watching them.

Rural; English-speaking participant; FG RE2

I also think it’s a very good idea because someone in my family has asthma, so, maybe that would have saved me a lot of sleepless nights.

Urban; Spanish-speaking participant; translated; FG US2

Others spoke about the value of mHealth monitoring for older people living independently:

This scenario made me think of my grandma who lives by herself, and she’s very much a lot older. And she has a lot of health issues, right? So I think if her pressure is low or something and she falls, that could be—I think [an mHealth device] would be really helpful.

Urban; English-speaking participant; FG UE1

I have a neighbor who lives alone and is already old, and that makes me have all the access of her movements. Yes I would like that device to monitor...Because nobody is aware of her, she is left alone, and I think it would be very helpful.

Rural; Spanish-speaking participant; translated; FG RS1

In addition, some discussed the role of mHealth in allowing people to continue their regular activities, sometimes referencing our slideshow example of the boy using mHealth to manage his asthma while continuing to play soccer. A participant highlighted the importance of allowing a child to play sports without the added burden of fear about asthma:

I think it is good because the child would not abandon the games that he likes. And if that [mobile device to track asthma] did not exist, a mother would say, “You better not play.” And if it did exist, she would say, “Yes, play. I am going keep an eye on you.” Then, the child will no longer say, “If I do this, this will happen to me.”

Rural; Spanish-speaking participant; translated; FG RS2

#### Theme 2: Some Are Unaware of, Unfamiliar With, or Uncomfortable With Technology and May Benefit From Individualized Support

Many participants noted that they had used mHealth apps for tracking things like physical activity or diet, but overall, most expressed a general unfamiliarity with mHealth technologies used to track and make predictions about risk for specific conditions. Several mentioned that no health professional had ever made them aware of such devices:

Well, I think [it would be good] for them to let us know that they have this kind of device, because I never heard that they have this device. They never told me.

Rural; English-speaking participant; FG RE2

[I would have questions about] the name of the devices, which company can provide them, if there is any support to be able to have those devices, if they are going to start providing them in the clinics, where one can get more information, or for example, in the clinic, who can one address specifically to say, “Hey, do you know if the device is already available”.... In other words, how can one go about obtaining these aids.

Rural; Spanish-speaking participant; FG RS2

Participants further noted that some people might feel uncomfortable with using technology. They cited examples of older family members either avoiding technology in general or specifically avoiding wearable health devices. Some used these examples to highlight how the value of a device is contingent on an individual’s comfort. For example, a participant provided an example of how their family supported their mother in getting comfortable with technology despite hesitancy about wearables and challenges with mobile devices:

So I know my mom wouldn’t wear one, and we always worry about her also. She’ll lose her phone and stuff, and we’ll be trying to call her, and she won’t find her phone, and so my best thing was to get her [a voice-activated device]. So I showed her how to use that, and she’s pretty okay with it. So it’s nothing she has to wear, but whenever she needs to make a phone call and she can’t find her phone, that’s what she uses.

Rural; English-speaking participant; FG RE1

Some participants expressed that most people could get used to new technology over time. One highlighted how quickly this could happen for children:

They just hop onto everything, whatever, and technology is not very difficult for them to get used to and learn it, and they pretty much get used to stuff real quick that has to do with—and especially if it has to do with their health is like you need to do it.

Rural; English-speaking participant; FG RE1

Others emphasized the need for practice over time:

Even if they want to, if it is not being used, they have the training, the direction—whatever they want to call it—to be able to use it and know what is going to happen, to know about the program or the equipment so they can help. If we don’t know how to use something, it doesn’t help us at all, on the contrary, it will give us more problems.

Urban; Spanish-speaking participant; FG UE3

I don’t think it will be hard to learn. It would just take some time. To get used to anything, you get used to a new phone, you get used to anything you get new. So you get used to it. So it takes practice.

Rural; English-speaking participant; FG RE2

Some participants also identified a need for individualized support, such as developer-created tutorials or one-on-one support, to facilitate comfort with mHealth. One participant discussed the value of personal support:

I think, especially for Latino families, I think we need more person-to-person support. Like, “Take this, read it. If you have any questions, find me, call me.” Because yes, sometimes we bring things into the house and we don’t understand it and we don’t know who to ask or how to use it. So, having that reinforcement, having that person who can guide you, help you and get you out of doubt when using new things.

Urban; Spanish-speaking participant; FG UE3

#### Theme 3: Financial Barriers Limit Access to mHealth

The cost of devices was a concern for many participants, who raised questions about whether devices would be covered by insurance or what the cost would be for those without insurance coverage. Several called out the likely or actual cost of these devices as a barrier:

It’ll probably come with a hefty price tag.

Urban; English-speaking participant; FG UE1

I haven’t used [mHealth], as the price is a bit high.

Rural; Spanish-speaking participant; translated; FG RS2

I had one that was given to me as a gift, but it was one of the first ones that came out. Then, after that, well, I didn’t—the truth is, financially I never put money to buy a new one for me, because they were more sophisticated, more expensive.

Urban; Spanish-speaking participant; translated; FG US2

Some participants specifically noted the potential expense if multiple family members needed their own devices:

I would imagine for those who have more than one person in the family, it will cost a lot of money.

Rural; English-speaking participant; FG RE2

If there are three, four, five children in the family, it is not enough to be buying—we are talking about the economic [impact], to be buying devices of all kinds and that [have] everything the child needs.

Rural; Spanish-speaking participant; translated; FG RS1

Several participants highlighted the importance of insurance coverage for affordability, including how inconsistent insurance coverage could impact people’s use of mHealth:

[MHealth devices] are expensive and all this. But if you have insurance and if it's within the insurance budget, they’re not expensive.

Urban; Spanish-speaking participant; translated; FG US2

The cost, that would be a problem that many people face, such as the costs of the products that provide us with these types of services. Like, for example, in my case I use a sensor, if it were not because I have an insurance, I couldn’t afford it.

Urban; Spanish-speaking participant; translated; FG US1

I think it also depends on cost or insurance, because a lot of us don’t have access. An example. Right now, I no longer have access to insurance since I stopped working, because I have a temporary job, so in two months I run out of insurance. So, that’s when the benefit comes or we no longer have benefit, for the drugs, the appointment, everything that comes with it is more expensive. So certain people who have or do not have health insurance, that’s where I think the conflict really comes. But I think a lot of people would be willing to use it if they are cheap.

Rural; Spanish-speaking participant; translated; FG RS1

One participant expressly noted that these resource limitations may disproportionately affect Latino individuals:

And of course, always the Latinos, right [laughs], we don’t have the resources of those who speak English...Well, yes, the whites, they have them, we don’t have them.

Urban; Spanish-speaking participant; translated; FG US2

#### Theme 4: Practical Considerations Create Barriers to Using mHealth in Daily Life

Participants also discussed potential technical barriers, including inconsistent internet access, limited phone storage capacity, limited battery life, and technology crashing or simply not working. For example, a rural participant commented on the challenges of relying on inconsistent internet and cell service in their area:

How many times does our internet go away? How many times do the poles get knocked down or whatever, or there’s lost connections? Something that we can have, like maybe going back to those dial tones. I don’t know. Plugging them into the wall. I don’t know, but trying to find something that’s not going to crash on us, especially if your child is having one of these asthmatic attacks.

Rural; English-speaking participant; FG RE2

Other participants also commented on inconsistencies in internet access:

Not everybody is going to have access to it. As they say now, “The Internet,” but there are still places and there are still people who do not have access to it.

Urban; Spanish-speaking participant; translated; FG US3

The only problem would be if, for example, how the device works; if it is with electricity, and if one day the power goes out; or if it is by means of wifi, even if the power goes out, it stops working.

Urban; Spanish-speaking participant; translated; FG US2

Participants also described the breadth of challenges that people experience with their mobile devices:

Every device can fail at some point, or maybe it is low on battery or I don’t know what it will have or—there is always a problem, but there is always—nothing is 100% safe.

Urban; Spanish-speaking participant; translated; FG US1

The charge doesn’t last long. So maybe having something that doesn’t die so quickly. Again, mine could be because it’s an older version or maybe it’s time. Maybe it’s a hint of having to go get a new one. I don’t know. But the fact that it dies really, really fast because it’s calculating everything. The more apps you have on it, the more battery it takes.

Rural; English-speaking participant; FG RE2

It adds very quickly to your memory, it fills up very quickly. Well, make it less. Because can you imagine if we have a disease app? People who sometimes have so many diseases are going to fill up the phone and sometimes the memory is not enough for them. Let it be less, please.

Rural, Spanish-speaking participant; translated; FG RS2

Participants further noted that the benefits of mHealth would be limited if not embedded within an environment that facilitated those benefits. For example, a child using a smartwatch to manage their asthma would need their school to allow them to use the device and coordinate with the family:

I wanted to ask you, to see if this device can be taken to school and the teacher can also have the information about the child, to be able to help him, because, just as the other family members can also help, they will have the information, the teacher can also help you.

Urban; Spanish-speaking participant; translated; FG US1

Similarly, another participant noted that not all work settings facilitate the use of mHealth devices:

Sometimes at work you can’t even see the clock, much less the telephone. So, I think it would be a little difficult.

Urban; Spanish-speaking participant; translated; FG US2

#### Theme 5: mHealth Raises Concern for Overreliance on Technology

Some participants raised concerns about mHealth leading to overdependence on technology. For example, one participant described mHealth as a part of a broader societal move toward technology dependence:

Yes, we are advancing and we are advanced, but they are the things that leave us like a bad taste in our mouths: we are not up to date in the moment, in the second. Because I saw that suddenly, “Oh, three more hours on my phone. Leave me, I reinstall it, because it is not up to date.”

Rural; Spanish-speaking participant; translated; FG RS1

Another participant discussed how technology could undermine one’s self-reliance, especially for a child:

One of the challenges that I noticed even in the cartoon characters, that they showed Arturo’s discouragement and feeling like he’s trapped in all this technology causing him to not be the person that he wants to be because he wants to be a good soccer player, for sure. So that could interfere with his psyche and his confidence in himself.

Rural; English-speaking participant; FG RE1

Others spoke about the potential impact on family relationships. Some raised concerns about family members relying on devices and consequently neglecting to engage directly with their family members about their health:

I think there is a lot of elderly very, very alone, and this will make it worse, like, “Oh, okay. It will let me know. The little thing will send me a message if she falls.” And I won’t be able to like it. I’m kind of more old fashioned. I think maybe I can get more involved or ask a friend or ask more family to take care or be a company [] instead of just put a collar on it and just forget about it.

Urban; English-speaking participant; FG UE1

I have a teenager, and sometimes it’s hard to make a conversation with them, right? So I think it will be more challenged to connect with them if you’re just looking at the phone instead of asking him, “How you feel during the day?” or, “How was the challenge? How you feel when you run and jump?” And if I’m just worried about the app or what the phone said or the wrist coughing track, for me, it would be like I don’t care how—it’s going to be like stopping the communication. I would be more worried about the monitor and not asking my child. And maybe they will feel like, “Oh, she knows now that I’ve been coughing because she’s been monitoring,” instead of telling me, “How you feel with this and that?” And maybe the monitor is tracking just the coughing, but it didn’t tell me what activities they were doing, and I think we’ll be much better to stay with one-on-one communication with our kids.

Urban; English-speaking participant; FG UE1

#### Theme 6: Automated mHealth Features are Perceived as Valuable but Fallible, Requiring Human Input to Ensure Accuracy

Limitations of technology also came up in terms of data collection. Although automatic, continuous data collection was often seen as beneficial and easy, some participants noted that manual input would be more reliable in some cases, most notably in tracking mood and mental health. One participant suggested that manual input for a period would improve subsequent accuracy:

You wouldn’t want or you wouldn’t be able to just be with the child 24/7 all the time, which is why automatic kind of makes sense in this really busy world. But I guess in something like this where it’s life or death and it’s your child, you’re willing to drop everything to be with them until this gets settled. So whether it’s the day, the week, the weeks to then get it honed down to the accuracy and the level of punctuality that you need, it would make sense to do it manually as well. And then work automatically.

Rural; English-speaking participant; FG RE1

Another highlighted an example from their own experience of needing a human to step in when health technology fails:

I think we are going to realize if something is wrong with the device or with our child. I tell you this because my grandson uses a feeding pump and when the pump is failing, we can tell. So, then we call the technician and we have the possibility to feed him manually.

Urban; Spanish-speaking participant; translated; FG US3

Others discussed the importance of checking on data from the device to verify the information it provided, viewing the role of the device as providing them with a high level of information and then allowing them to make decisions. For example, one participant described the need to balance the ability to take action when warranted with avoiding panic due to frequent alarms:

Imagine if [the device] is alarming every hour, then that can cause not only that the person gets scared or the child gets scared, but the others stay in panic, because they don’t know if it is right, if it is correct. So it would be like, maybe, give them time to be able to verify all the symptoms and that yes, instead of sending and sending alerts.

Urban; Spanish-speaking participant; translated; FG US3

Another expressed skepticism that the information would provide clinical value:

For me, when my kids get sick or something, I never been asked by the pediatrician how many times it was coughing, never had to track that. It’s just in general, how the kids are eating or playing or how they look, their appearance. Sometimes they look very tired, or you can tell when they couldn’t breathe very well...So I think the monitor will be hard to track that, and I won’t trust completely like, “Oh, you coughed 100 times. Let’s go to the hospital.”

Urban; English-speaking participant; FG UE1

Despite these concerns, participants generally indicated that they would prefer to receive false positive alerts rather than miss true positives:

I think, for me, it would be more stressful if I didn’t detect a real asthma attack. Since it’s about saving the child’s life, what does it matter if it’s a false alarm? Well, yes, I’m going to be a little stressed, but I’d rather have that than it not detect an asthma attack. I know that computers can’t replace humans and they’re not perfect either, but yes, I’d rather have the device be more sensitive and have a false alarm once in a while than not detect, not be sensitive enough to detect all asthma attacks.

Urban; Spanish-speaking participant; translated; FG US2

I would rather have a million false alarms than the one where they direly needed help and that one wasn’t alerted.

Rural; English-speaking participant; FG RE1

#### Theme 7: Data Sharing Is Seen as Valuable for Limited Uses but Raises Privacy Concerns

Overall, participants endorsed the value of data sharing for uses such as providing emergency support or sharing clinical information with key family members or clinical team members. Participants also supported the use of data for improving the technology and making better predictions, at least on the level of the individual device. For example, one person described the importance of collecting location data as well as health data, both for emergency services and to improve accurate predictions:

I think it’s very good, because it is giving the place where he is at. And let’s say he is feeling very, very bad and can’t even talk, then all that helps. And if someone is watching him, it would help, if he is in a place where no one else is around or close by, to find him, to alert the doctor, to know that the child is going to need help or that he is in difficulty...And maybe they can add more information, like “where you are, you’re prone to have really bad air at this time of day.” All of that could help them, the kids, make the best decisions for themselves.

Urban; Spanish-speaking participant; translated; FG US3

However, another participant described data sharing as a “double-edged sword” that would increase privacy concerns:

I know that it is to improve the service or the product, but it is also like a double-edged sword, because your information goes into more hands so, if it is no longer completely clear to you how your information is handled, when it is already in the hands of other people. So, in that aspect, I would like to have a little more privacy.

Urban; Spanish-speaking participant; translated; FG US1

Other participants also identified privacy concerns, highlighting the possibility of data breaches or data misuse:

So if somebody who isn’t a professional hacks into his watch, that would be invasion of his privacy. If his friends, people at school, hack into his watch, he would probably be embarrassed. And again, it’s just taking away his privacy, which the hacking is—could be an issue.

Rural; English-speaking participant; FG RE1

I would be afraid that, let’s say, the medicine companies might say, “In this area there are many children with asthma or more attacks” and they might want to take advantage of that situation in some way. I don’t know. And also, since all the information is virtual, and even if one is careful and cautious, there are many places where one’s private information has been seen by many people who have nothing to do with it.

Urban; Spanish-speaking participant; translated; FG US3

These concerns were heightened for information they deemed sensitive, such as location data and information about sensitive or stigmatized health conditions. One participant identified mental health as an area that might raise particular concerns:

I think the disadvantage of using these technologies for more private things, as a mental health issue, is that there are times that they do like hacking, that they get into your phone and grab information that, obviously, you don’t want other people to know or that is very sensitive. That’s why I sometimes get nervous when I’m putting medical stuff on my phone.

Rural; Spanish-speaking participant; translated; FG RS1

Participants also suggested ways to find a balance between the value of data sharing in limited cases and the risks of privacy breaches. For example, one person proposed limits on the types of data to be shared:

Sometimes, everyone wants to keep [their information] private, and even more so when it comes to children, so maybe you are supposed to be sending the information to trusted people, but, for example, as a mother I would feel safer just having my daughter’s location. So, or her father, as [another participant] mentioned. That is, the people as closer. But from then on, like, for example, the activity of coughing or anything else, obviously it does have to be shared, but for me, the location [should not be shared].

Urban; Spanish-speaking participant; translated; FG US1

Another suggested that those accessing data enter into explicit agreements about data use:

The only ones that have access to the location would be his doctor or his doctor’s office or the emergency room, the ambulance. I think that that would be—I know as a parent myself, I would be scared about people knowing where my kids’ locations is at, right?...But giving access just to— even signing something saying that they’re the only ones that are going to have access to their location in case of an emergency. Something written, not verbal. I would get something documented, written, that says, “I am only going to have access to their location in case of an emergency.”

Rural; English-speaking participant; FG RE2

## Discussion

### Principal Findings

Our focus group findings report important perspectives on mHealth from Hispanic and Latinx individuals across rural and urban regions of Washington State. Although the themes we report share similarities with perspectives of other communities, they highlight the importance of tailoring mHealth tools to the context in which they will be used, specifically among a community with high rates of underinsurance and limited financial resources, including many individuals in rural areas and many whose primary language is Spanish. Our results illustrate key barriers to the benefits of mHealth in these communities, provide insights into the role of mHealth within families, and examine the appropriate balance of data sharing and privacy protections.

### Barriers to the Benefits of mHealth

Participants perceived mHealth as beneficial overall; however, our findings highlight several critical barriers to accessing those benefits. First, there was little awareness of the potential for mHealth to be used to manage chronic conditions. Participants with experience with asthma or other conditions expressed particular interest in having a health professional tell them about these types of tools. Second, many participants expressed that they or their family members might be uncomfortable using mHealth technologies, which could lead to them either never initiating or stopping the use of a device. Suggestions to provide time for practice, tutorials, or even personalized support arose in multiple focus groups as strategies to improve comfort and familiarity. Third, participants reported resource constraints and the lack of insurance coverage as significant barriers to accessing mHealth. Finally, participants identified both features of the device (eg, technical usability) and broader contextual features (eg, integration with school systems) as limiting the usability of mHealth tools if not adequately addressed. These findings, particularly related to resources and provision of support, are mirrored in qualitative work with other populations, including residents of rural areas [35] and English- and Spanish-speaking patients in a safety net setting (ie, care providers that serve patients regardless of their insurance status or ability to pay) [36]. Another pilot study that included interviews with 10 Latina women similarly identified limited access and underutilization of mHealth as limiting the technology’s social value to the broader community [37]. The imbalance of high perceived benefits with significant barriers illustrates the need to approach mHealth research and development from a justice-oriented perspective, as Herington et al [24] argue in the context of digital health research that works on both decreasing risks and, importantly, developing specific, community-informed mechanisms to equitably share benefits.

### The Role of mHealth in the Family

Participants also discussed the complex role of mHealth within family dynamics. Many identified relational benefits of using mHealth devices to make personalized predictions about risk when used in a family setting, providing caregivers of children and older adults with peace of mind. At the same time, participants acknowledged that their family members, particularly older adults, may view such monitoring as interfering with their autonomy. Some also worried about the risk of undermining interpersonal relationships from relying too heavily on technology. These dynamics reflect the value of considering a “family informatics” approach to mHealth design, which explicitly recognizes the interrelatedness of family members and their health [38]. Family connectedness and commitment are core cultural values that may shape how Latinx individuals approach their use of mHealth devices [23,39]. Thus, family informatics may be particularly relevant for ensuring devices are culturally appropriate. In addition, to navigate concerns for overreliance on technology, developers should consider hybrid solutions that integrate the benefits of automated technology alongside human input [40].

### Data Sharing and Privacy Concerns

A key issue in the use of mHealth is how data are shared, including whether and how artificial intelligence incorporates user data [41]. In discussions of data sharing, participants identified specific benefits in the use of data sharing to facilitate emergency responses and improve personalized prediction accuracy. Although disclosing individual data was generally seen as acceptable for these beneficial uses, this was balanced with privacy considerations, particularly around location data and sensitive health information. Privacy concerns and the risks of breaches of privacy may be heightened among individuals from marginalized groups, who differentially experience harmful impacts from discriminatory social structures [24,42]. Primary language is one relevant dimension of marginalization; a recent survey of parents of children with asthma found that Spanish-speaking parents were more likely than their English-speaking counterparts to report privacy concerns about digital health tools [17].

Participants suggested solutions to limit data access to only those necessary to produce the benefits identified as valuable. Discussion about data sharing for purposes of improving predictions more generally was limited but similarly focused on benefits and risks. A key way to accomplish this appropriate balance will be to ensure that the benefits of artificial intelligence–enabled devices do, in fact, reach the communities that are taking on privacy risks [41,43]. Future work should continue to examine the nuances of community members’ perspectives on this benefit-risk balance in the context of predictive technologies.

### Limitations

Our study represents the views of community members drawn from a specific region of the United States; people with other life experiences, cultural, ethnic, or national backgrounds [44], and values may have different perspectives. Of note, most (43/48, 90%) participants were women, and most (35/48, 73%) reported that Mexico was their country of origin. Nevertheless, by situating our findings within the broader literature on community perspectives about mHealth and other health technology, we aim to provide nuance from the perspective of this underrepresented group.

Further, although we explored topics related to the use of mHealth by children and older adults, children were not eligible to participate in this study, and participants were in midadulthood on average. Additional perspectives would be important to include in guidance on devices targeted at additional age groups. Future work is also needed to explore mHealth as applied to specific conditions, such as mental health conditions that may raise additional privacy considerations.

Finally, while our case examples illustrated artificial intelligence–enabled mHealth tools and focus group discussions built on these examples, we were not able to fully explore the complexities of attitudes about artificial intelligence within this study. Future work should continue to seek ways to engage with community members about these topics and their implications.

### Conclusions

This focus group study with Hispanic and Latinx community members illustrates the importance of developing mHealth with input from underrepresented communities. The in-depth perspectives of these participants highlight the necessity of developing and implementing tools with attention to barriers, family context, and privacy concerns to avoid exacerbating disparities in the use and value of health technology. Future work should incorporate these perspectives into guidance to support mHealth developers in creating tools that meet community needs and advance equity.

## Appendix

Multimedia Appendix 1 Focus group guide.

Multimedia Appendix 2 Narrative slideshow (English version).

Multimedia Appendix 3 Narrative slideshow (Spanish version).

## Abbreviations

mHealth: mobile health

## Data Availability

The datasets generated and analyzed during this study are available from the corresponding author on reasonable written request.
