# Supplementary material for: Perspectives of Hispanic and Latinx Community Members on AI-Enabled mHealth Tools: Qualitative Focus Group Study
Source: J Med Internet Res. 2025 Feb 6;27:e59817. doi: 10.2196/59817 (PMC11843051; doi:10.2196/59817)
Supplement: Multimedia Appendix 1 [file jmir_v27i1e59817_app1.docx]

**Focus group guide**

**INTRODUCTION AND CONSENT (5 MIN)**

Thank you for taking part in this focus group. I’m [INTRODUCE SELF] and I’ll be leading our discussion today. Also here are [INTRODUCE OTHER TEAM MEMBERS AND ROLES].

Our goal today is to learn what you think about different technologies that are available, or that might be created, to help people to track their personal health. This includes things like devices you might wear to track things like your heart rate or your exercise habits, as well as the applications you might use to look at the information or track other things like your sleep or diet. As we talk, we’ll share some specific examples of these technologies to give you a better idea of what these could look like and how someone might use them in their daily life. We’re interested in hearing what you think, as well as what your family, friends, or community members might think about these, including the positives as well as the negatives.

Before we get started, I want to share a few things about how our discussion will go. First, there are no right or wrong answers, and it’s okay to disagree with a perspective someone else shares. We just ask that everyone remain respectful. Second, please try to stay on mute when you’re not talking. This will make the sound quality best. You can unmute to show me you’d like to say something, or jump in if there is a pause in conversation. I’d like to hear from everyone as much as possible, so I may call on people if I haven’t heard much from you. You’re always welcome to pass.

Finally, we would like to record this session so that we can have a good record of our discussion. To preserve everyone’s privacy, please try to avoid sharing identifiable information, like names of people or places, about yourself or others. However, if anything is said that might identify someone, we’ll remove that information before we share the results outside of the study team. At this time, I’d like to ask each of you to confirm that I have your permission to turn the recording on. [CONFIRM PERMISSION AND TURN ON RECORDING]

Everyone in the room has given permission to be recorded, and the recording has now started.

Does anyone have any questions or concerns before we get started?

**WARM-UP (5 MIN) *[note to facilitator: use this question to try to get everyone to talk, even those who haven’t used one of these devices]***

1. I’d like to start by getting a sense of how, if at all, you have used technology to track your health or wellness. For example, maybe you use an app on your phone to track your sleep or your diet. Or maybe you’ve used a Fitbit or another wearable sensor to count your steps or track your heart rate. Has anyone used any of these, or something similar?
   1. [if yes] Which of these have you used? What do you like about it? What don’t you like about it?
   2. For those who have never used one of these technologies, what do you think about them?
      1. [if needed] Is there a reason why you do not / have not used such technologies?
      2. [if needed] Is there anything that might make you want to use one?

**EXAMPLE 1 (30 MIN)**

Thank you for sharing your responses. Now I want to show you an example of how technology could be used to track a person’s asthma. I’m going to show you a slide show about this example, then I’ll ask some questions to hear your reactions to it.

***Scenario 1: Maria and Sofia [refer to slide deck]***

1. What questions do you have about this example?
2. Imagine you had a child with asthma. How would you feel about using a cough monitor like the one in this example?
   1. [if needed] How do you think other people in your family or community would feel about this? For example, your parents or neighbors?
   2. How would your child feel about using it?
   3. What would you want to know from your child’s doctor about this?
   4. What problems do you see with using this?

Thank you for sharing your reactions. Now let’s look at another example to think about a few more questions.

***Scenario 2: Diana and Arturo [refer to slide deck]***

1. This example described three types of trackers: an air quality monitor, a smartwatch that Arturo wears, and an app that Diana uses. How easy or hard does it seem it would be to use each of these trackers?
   1. [probe if needed] What are some of the challenges you could see with using these?
2. This example showed an air quality monitor that would sit in one place as well as a smartwatch that could be worn on the body. What do you feel are the advantages and disadvantages of these options for the location of the tracker?
3. Any tracker can be wrong. For example, it might predict an asthma attack when there isn’t one or miss an asthma attack when there is one. How would you feel about it missing an asthma attack when there is one versus getting false alarms when there isn’t really one?

Thank you for your thoughts so far. Now I’d like to go back to our example and focus on how these technologies work and how they use people’s information.

***Scenario 3: Cough Monitors Machine Learning and Data Sharing [refer to slide deck]***

1. What are your thoughts about the different types of information that this technology might collect?
   1. How do you feel about how the information would be used?
   2. Are there types of information you would not want these tools to collect? Why?
   3. Are there any people or organizations you would not want your information shared with? Why?
   4. In this scenario, they were discussing different options for who should get alerts from the technology. What are your thoughts about who the alerts should go to?

Is there anyone the alerts should not go to? Why?

1. How do you feel about the trackers learning from the user’s information to make better predictions in the future?
   1. [if needed] What are the benefits of this? What concerns do you have?
2. Some of the trackers we’ve discussed automatically collect information, without the person needing to do anything extra. With other trackers, the person puts the information into the device themselves. What do you think about these different options?
   1. [if needed] What are the positives and negatives of automatically collecting information versus manually collecting information?
   2. [if needed] What differences in the kind of information collected might you expect between the two approaches?

**EXAMPLE 2 (10 MIN)**

Thank you for all your thoughtful responses so far. I’m now going to share a different example about how trackers could be used to prevent falls in older adults, and then I’ll ask for your thoughts on this one.

***Scenario 4: Falls [refer to slide deck]***

1. How would you feel about using technology to track and predict risk of falls?
   1. What do you see as the benefits of this type of tracker, compared to the benefits of the trackers we discussed earlier? *(previous examples: smartwatch cough monitor, air quality monitor, inhaler tracking app)*
   2. Are there any concerns that this example raises that didn’t come up earlier?
   3. Would you have different concerns about privacy in this example?

**GENERAL QUESTIONS/WRAP-UP (15 MIN)**

Thank you for sharing all your perspectives about these scenarios. I’d now like to ask you to think across all the examples we discussed and ask a few more general questions about these kinds of tools.

1. We talked about predicting asthma attacks in children and falls in older adults. Technology can also track a lot of other health conditions. These could include sensitive or especially private topics, for example mental health. What do you think we haven’t talked about that would be important for using mobile health tools for a more sensitive health condition?
   1. [if needed] What do you see as the advantages and disadvantages of using mobile health for a sensitive health condition?
2. Is there anything these tools should not do? Why?
3. Does anyone have other thoughts they’d like to add before we wrap up?

That’s all the time we have for today. Thank you for a wonderful discussion. We really appreciate your time and your input on this important issue.

We may also reach out by email in a few months with a draft of our findings to get your feedback on it.

[WRAP UP, STOP RECORDING, INCENTIVES AND CONTACT INFORMATION]
