# Supplementary material for: Perspectives of Hispanic and Latinx Community Members on AI-Enabled mHealth Tools: Qualitative Focus Group Study
Source: J Med Internet Res. 2025 Feb 6;27:e59817. doi: 10.2196/59817 (PMC11843051; doi:10.2196/59817)
Supplement: Multimedia Appendix 2 [file jmir_v27i1e59817_app2.pdf]

The Inclusive Technology for the Health of the Community (ITEC) Research Study

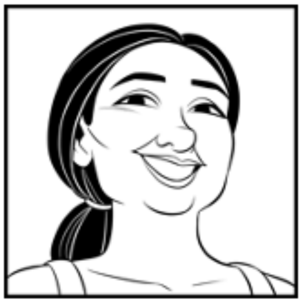

Maria

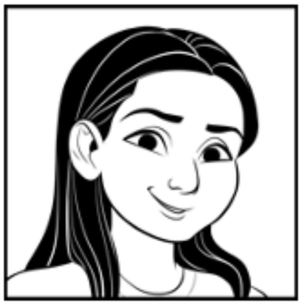

Sofia

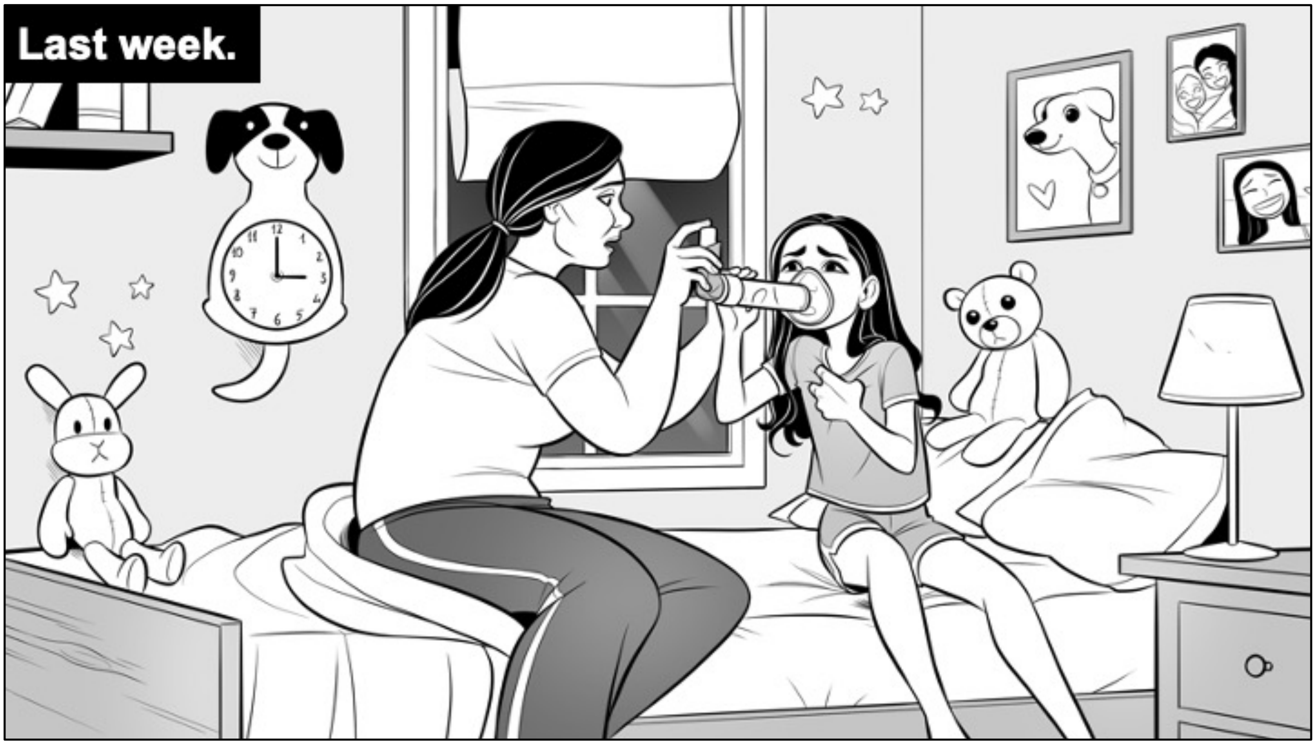

Focus group facilitator reads aloud: This is Maria and her daughter Sofia. Sofia is 10 years old and has asthma. Maria and Sofia live in an area where the air quality can sometimes be very bad. Last week, Sofia had an asthma attack in the middle of the night. She used her inhaler, but it did not help. She kept coughing and coughing and could not catch her breath.

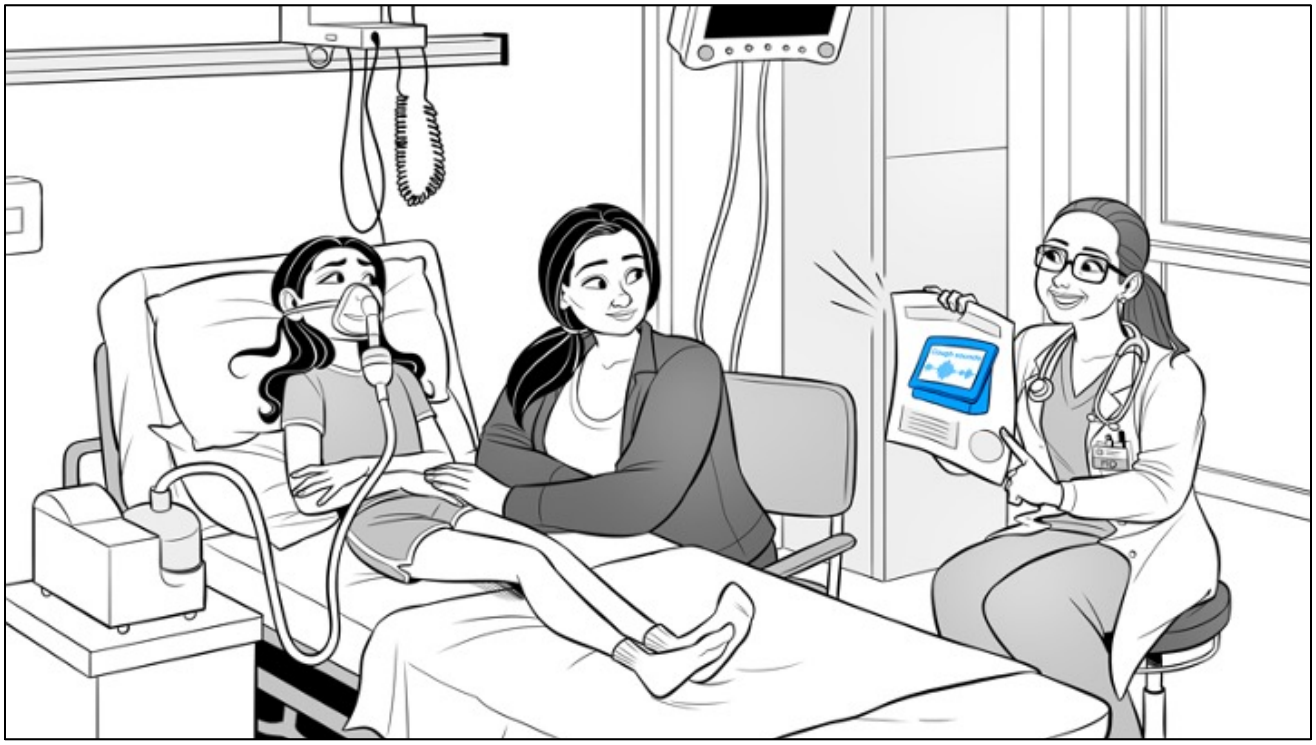

Focus group facilitator reads aloud: So, Maria took Sofia to the emergency room. After getting treatment for her asthma attack, Sofia was breathing comfortably again. The doctor recommended Maria consider getting a cough monitor for Sofia. Maria decides to get one.

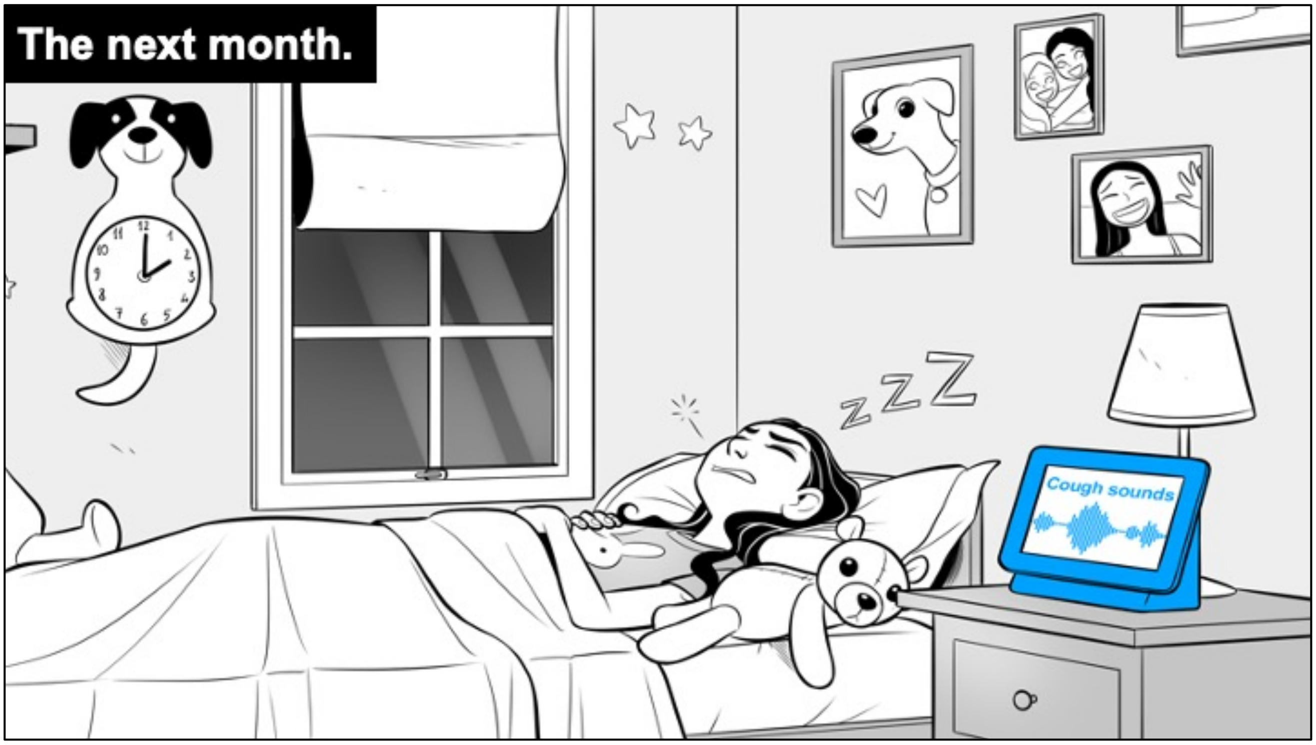

Focus group facilitator reads aloud: The next month, Maria sets up the cough monitor in Sofia’s bedroom. The cough monitor listens all night long. It records and checks any cough sounds. If it hears a lot of coughing, it can predict that Sofia might be about to have an asthma attack and will send an alert to Maria.

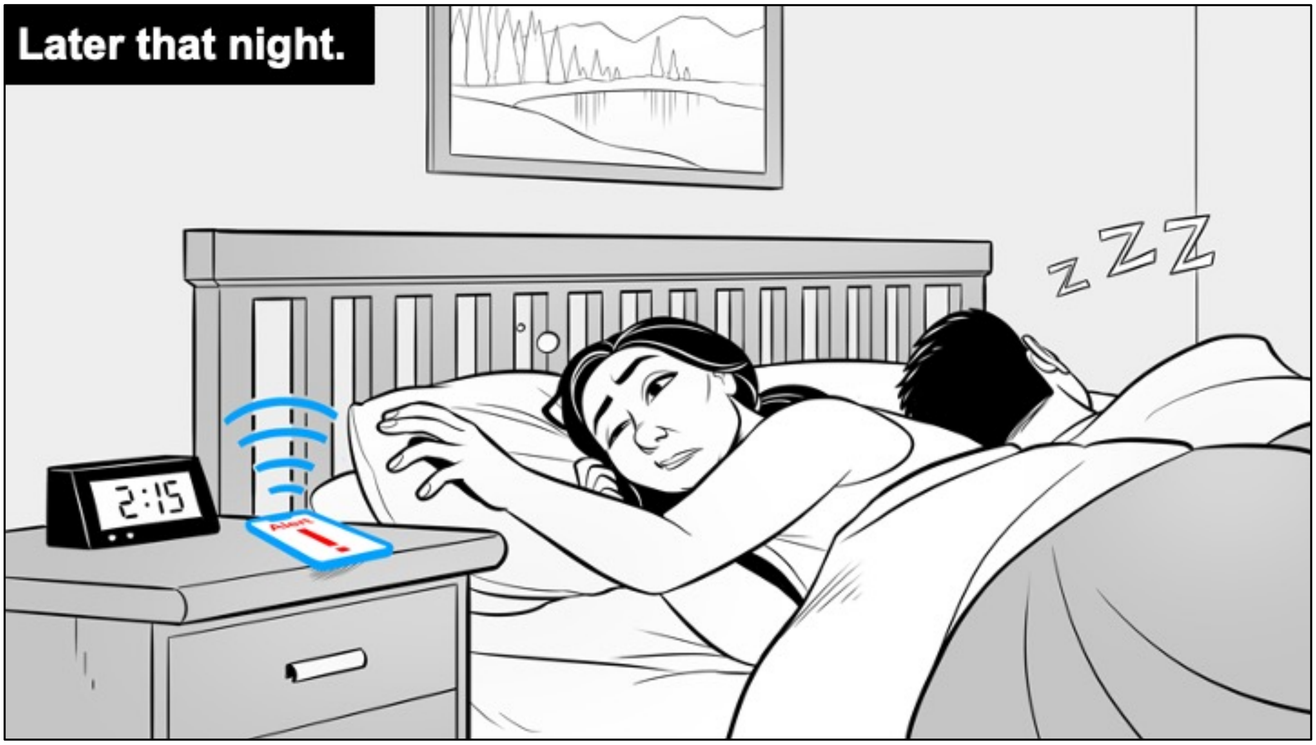

Focus group facilitator reads aloud: Later that night, the cough monitor detects that Sofia had started to cough a lot, so it sends an alert to Maria's phone. This wakes Maria up so she can help her daughter.

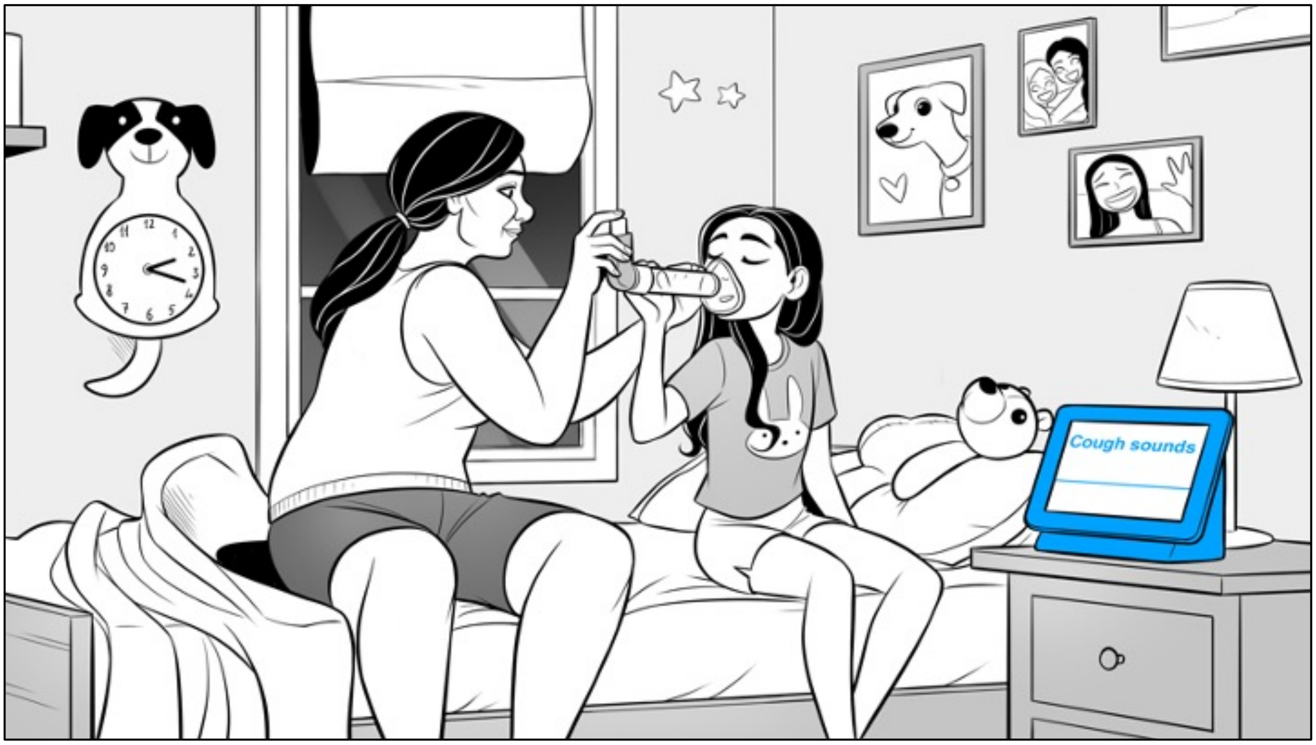

Focus group facilitator reads aloud: Maria helps Sofia use her inhaler right away. After taking the medicine, Sofia stops coughing, and they do not have to go to the emergency room.

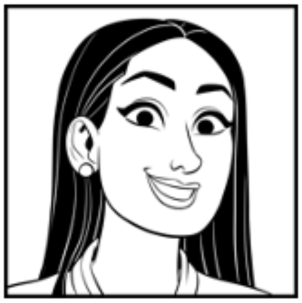

Diana

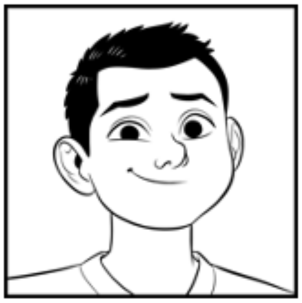

Arturo

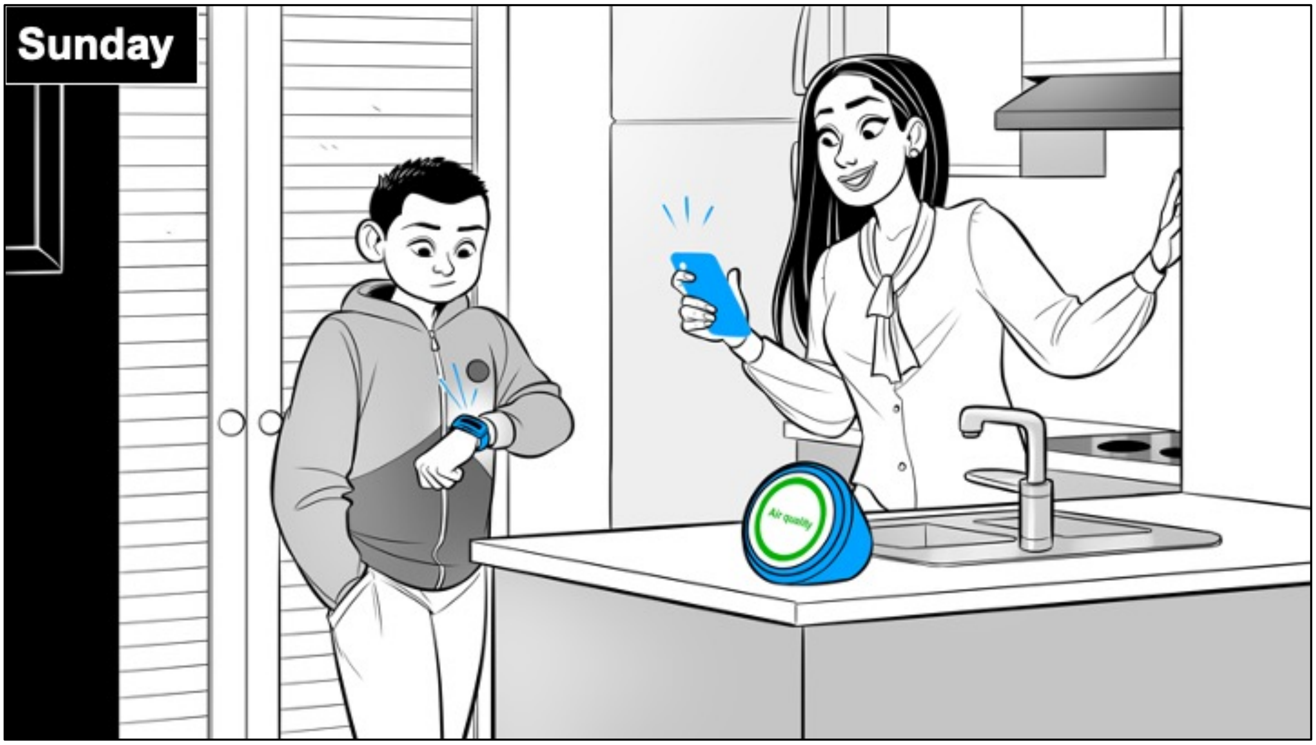

Focus group facilitator reads aloud: This is Diana and her son Arturo. Arturo is 13 years old and has asthma. Arturo loves soccer, and his team will play in the state championship game next week. Arturo wants to practice as much as he can before the big game. Diana's biggest concern is always keeping Arturo's asthma under control.

She uses some technology to do this

- An air quality monitor that shows if the air outside is unhealthy,
- A smartwatch with a cough monitor that Arturo wears every day,
- And an app on her phone where she can track each time Arturo uses his inhaler.

Like other technology, these devices come at a range of prices from more to less expensive.

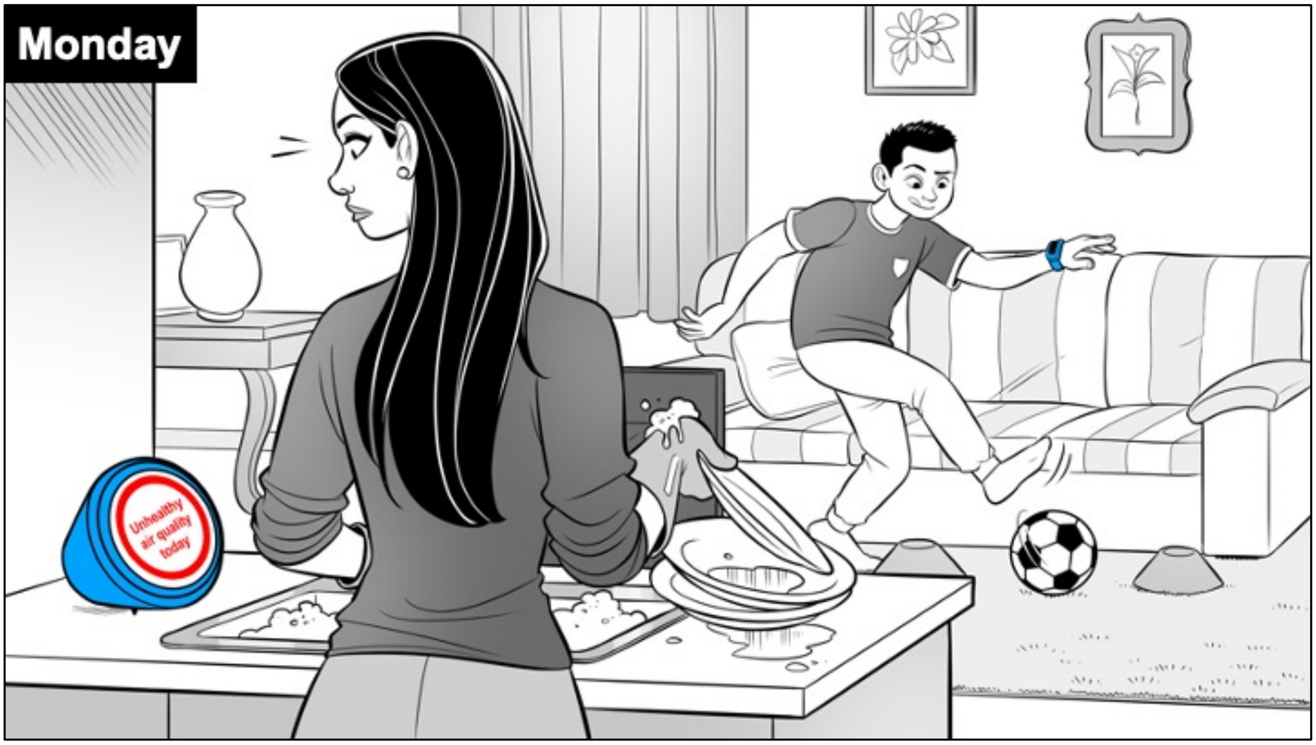

Focus group facilitator reads aloud: On Monday, the air quality monitor shows that the outside air is unhealthy today because of too much air pollution. Diana knows this is bad for Arturo's asthma, so she tells Arturo not to do his running exercises outside today. He agrees and does some soccer footwork exercises indoors to stay ready for the big game.

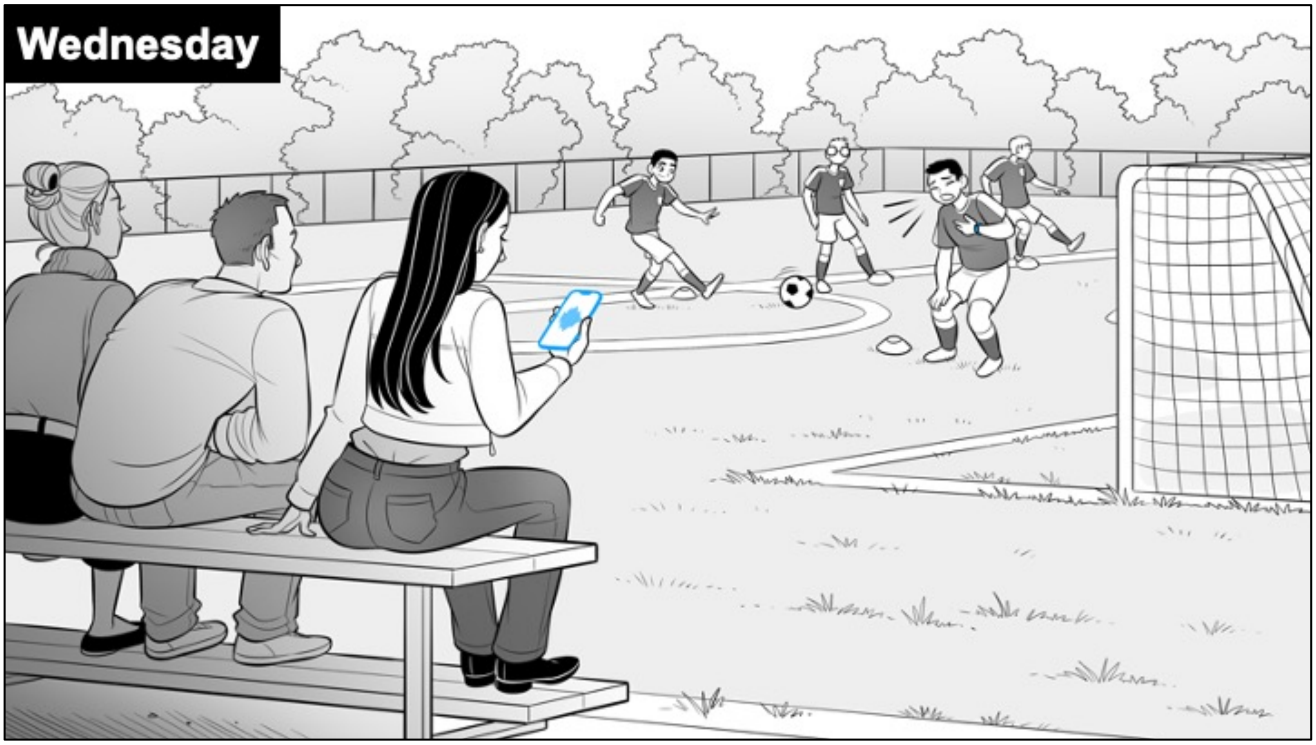

Focus group facilitator reads aloud: On Wednesday, Arturo goes to soccer practice. Diana watches from the sidelines. She checks the cough monitor app on her phone. The app shows Arturo has been coughing more during the night this week. She also notices that Arturo is coughing a lot during practice today, even though he already took his normal pre-exercise dose of his rescue inhaler. Diana brings Arturo his rescue inhaler for an additional dose.

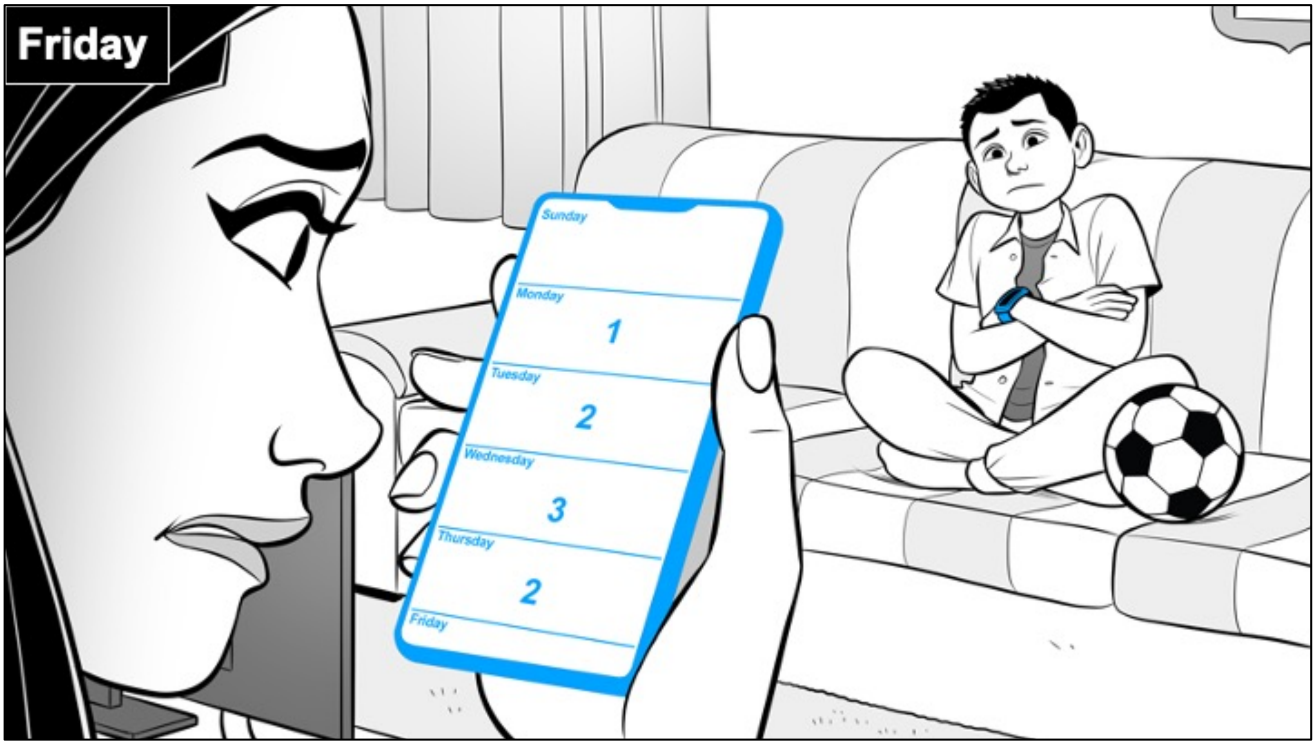

Focus group facilitator reads aloud: On Friday night, Diana looks at her inhaler monitoring app where she tracks each time Arturo uses his rescue inhaler. It shows that Arturo used his inhaler 4 times this week. She also knows Arturo has been coughing more than normal this week, because of the information from his cough monitor smartwatch. There are only a few practices left before the championship. Diana decides Arturo should not go to the practices. Instead, Arturo will stay home for a few days and use his asthma controller medicine more to help get his asthma back under control.

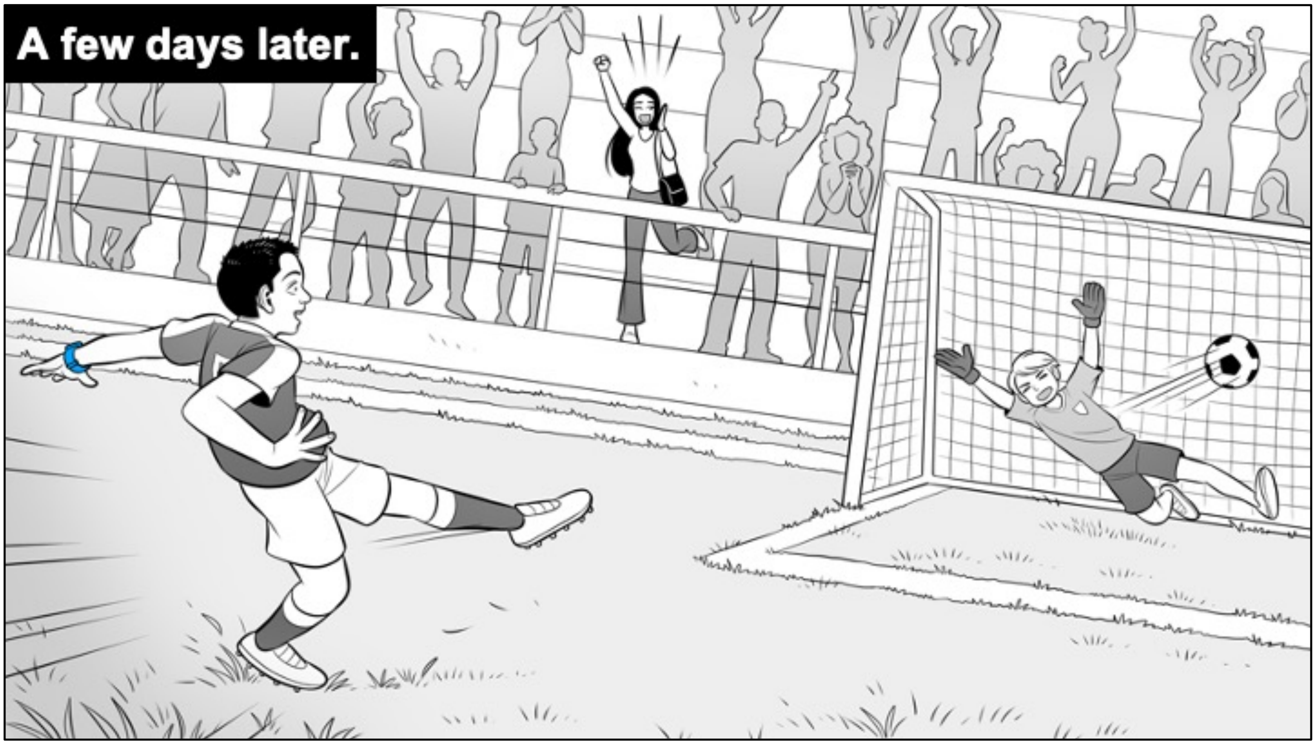

Focus group facilitator reads aloud: After Arturo sat out during the last few practices and took his asthma controller medicine, his breathing is better. Today, he can play in the state championship game. Diana feels good about her decision to keep him out of practice. She feels that if she had not made that choice, then Arturo might not be playing in the game today.

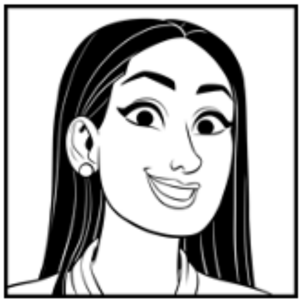

Diana

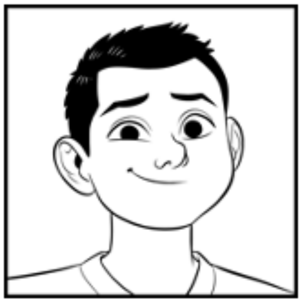

Arturo

continued…

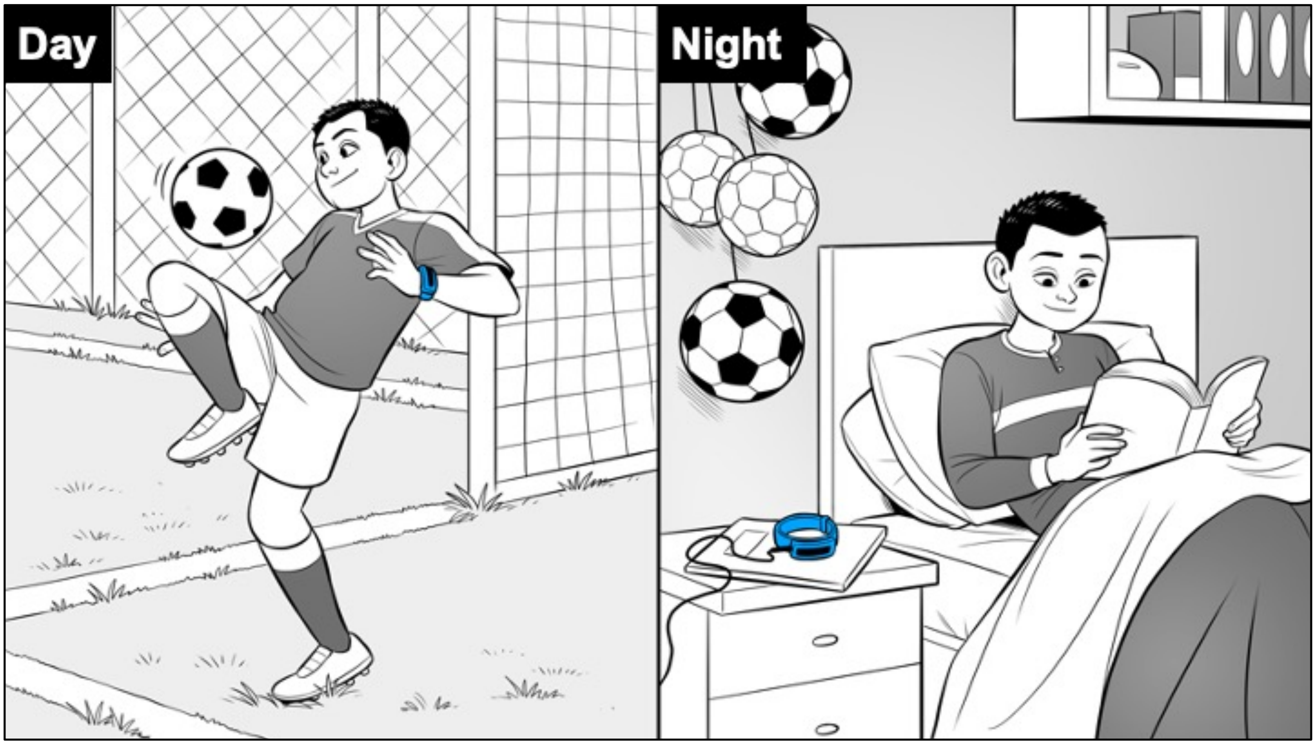

Focus group facilitator reads aloud: When Arturo first got his cough monitor smartwatch, it had to learn his normal cough patterns. He wore the watch all day and put it next to his bed at night. The app learned his daytime and nighttime cough patterns. This information helped the cough monitor learn when Arturo is coughing more than usual. This allowed it to learn to predict when Arturo is more likely to have an asthma attack.

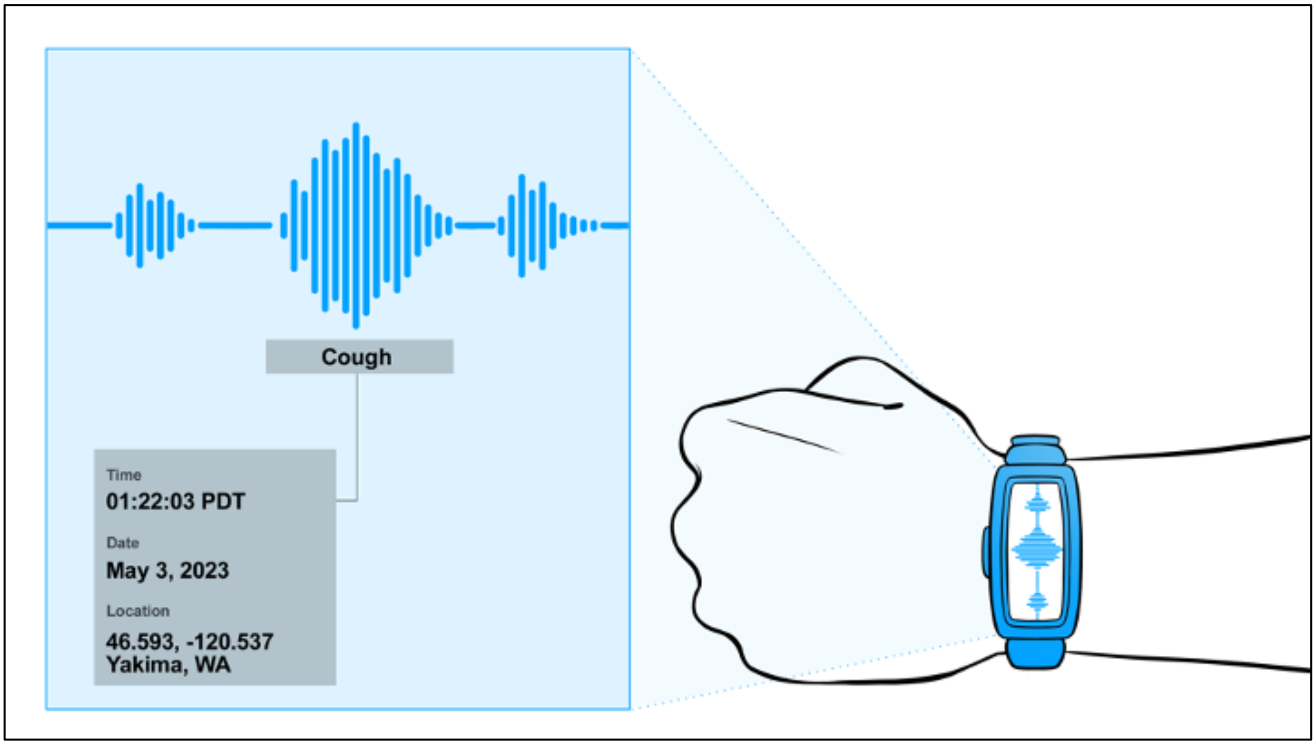

Focus group facilitator reads aloud: The monitor analyzes the sound of Arturo's cough and records his location. Cough monitors only detect coughing, not speech or other audio. When the cough monitor identifies that Arturo has been coughing more than usual and may be at risk of an asthma attack, it alerts him. But Arturo is not the only person who gets information from his cough monitor.

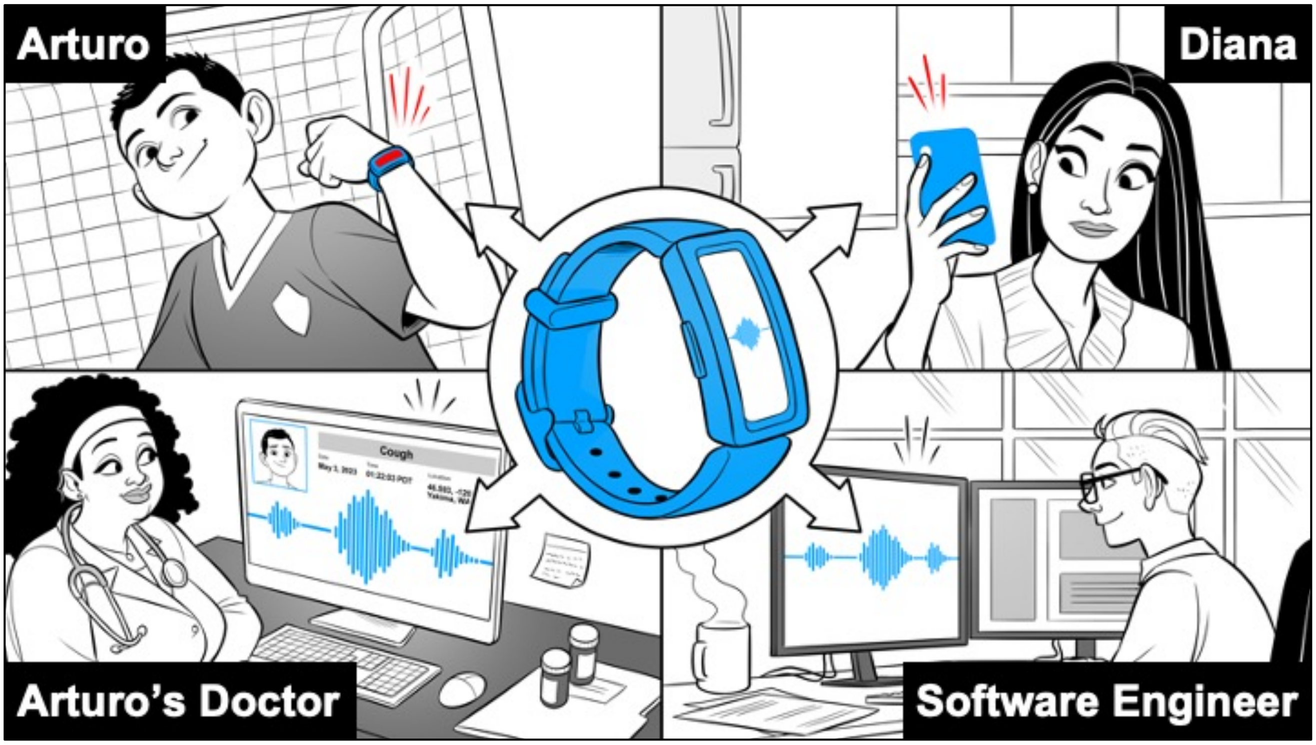

Focus group facilitator reads aloud: Here are all the people that get alerts and information from Arturo's cough monitor.

- Arturo gets alerts on his watch.
- Diana gets alerts and information on her phone.
- Arturo's doctor can also get information from the cough monitor. Diana set up the app to send the doctor the information the cough monitor collects, like the time and location of Arturo's coughs.
- The company that makes the cough monitor app also gets information. They get information from all the people who use their app. This information helps them make their app better. For example, sometimes cough monitors can have a false alarm –– they predict high risk for an asthma attack even when the person's asthma is under control. Using information from people who have their app, the company can update the app so it can do a better job at predicting asthma attacks.

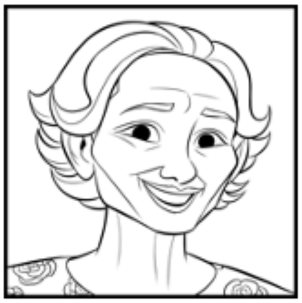

Dolores

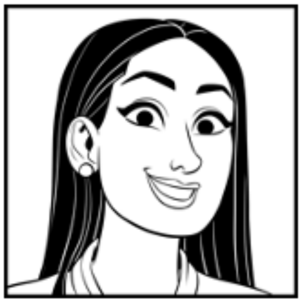

Diana

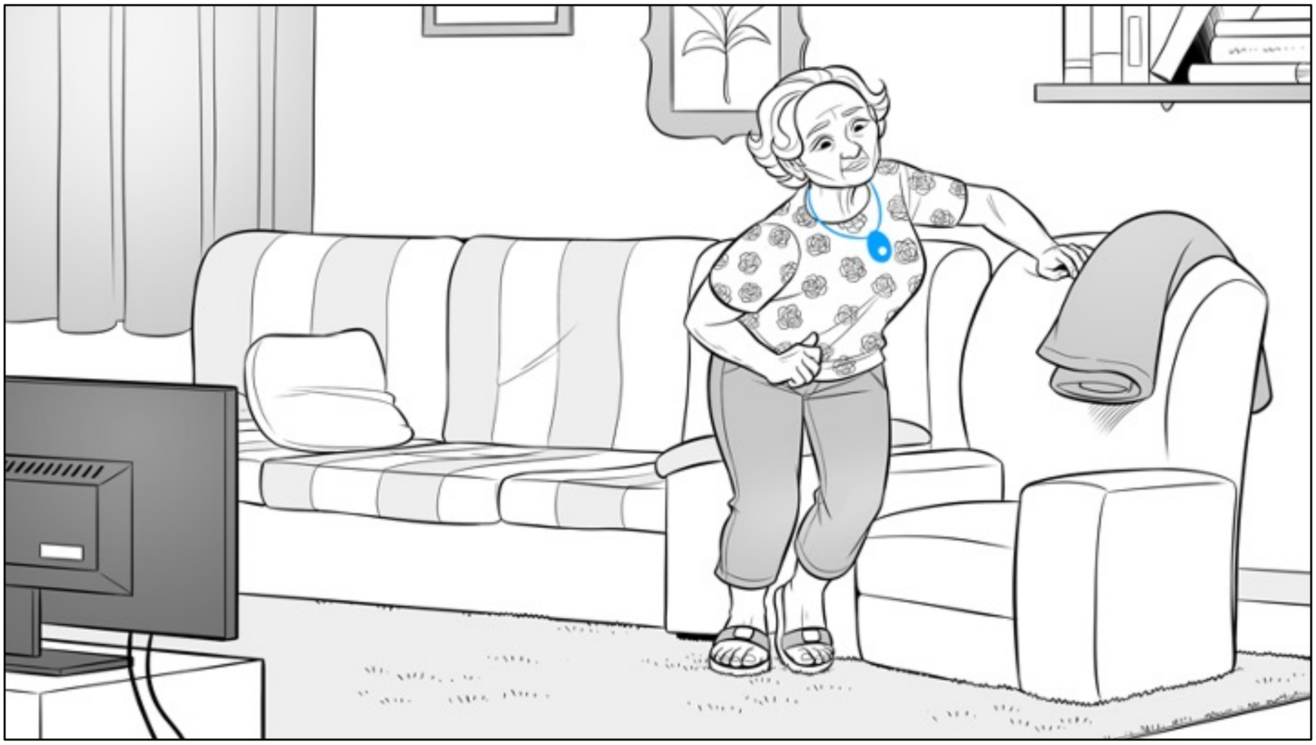

Focus group facilitator reads aloud: This is Diana’s abuelita Dolores. Dolores is almost 90, and she lives with Diana’s family. Diana is always worried that Dolores might fall because her balance has been getting worse as she gets older. Now that Diana has picked up an extra shift at work Dolores is going to be home alone for part of the day. So, Diana bought a tracking device that Dolores can wear like a necklace. It learns her typical movement patterns, so it can help predict when she is at risk of falling. If the device senses that Dolores is having trouble with her balance, it can send alerts to Diana and to Diana’s sister. If the device senses that Dolores falls, it can also send alerts to emergency services without anyone having to dial 911.
