# Supplementary material for: Perspectives of Hispanic and Latinx Community Members on AI-Enabled mHealth Tools: Qualitative Focus Group Study
Source: J Med Internet Res. 2025 Feb 6;27:e59817. doi: 10.2196/59817 (PMC11843051; doi:10.2196/59817)
Supplement: Multimedia Appendix 3 [file jmir_v27i1e59817_app3.pdf]

Incluyendo la tecnología para la salud de la comunidad (ITEC)

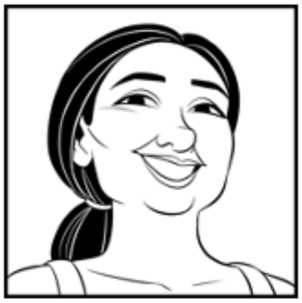

María

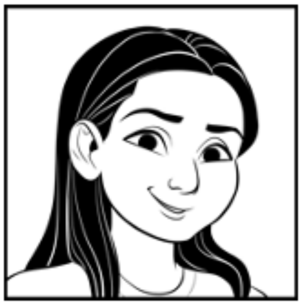

Sofía

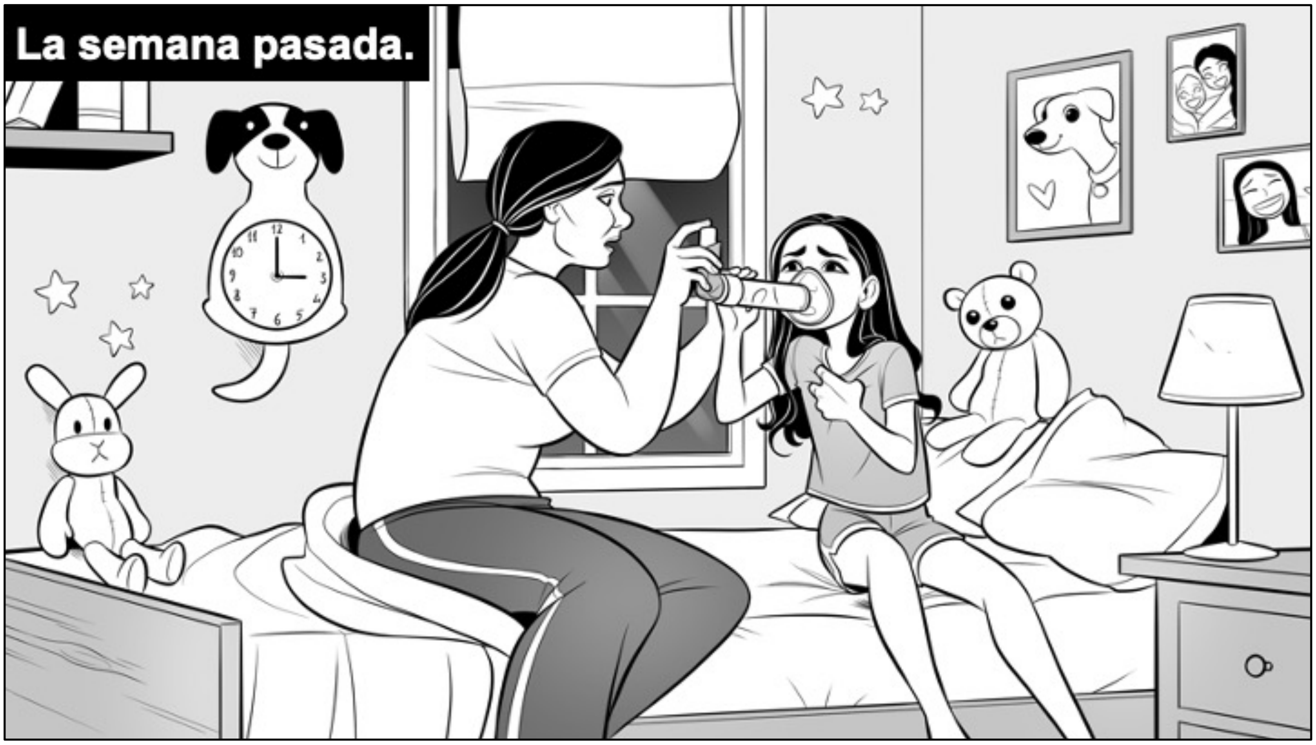

El facilitador del grupo focal lee en voz alta:
Ellas son María y su hija Sofía. Sofía tiene 10 años y tiene asma. María y Sofía viven en una zona donde la calidad del aire a veces puede ser muy mala. La semana pasada, Sofía tuvo un ataque de asma en la madrugada. Usó su inhalador, pero no le ayudó. Siguió tosiendo y tosiendo y no podía recuperar el aliento.

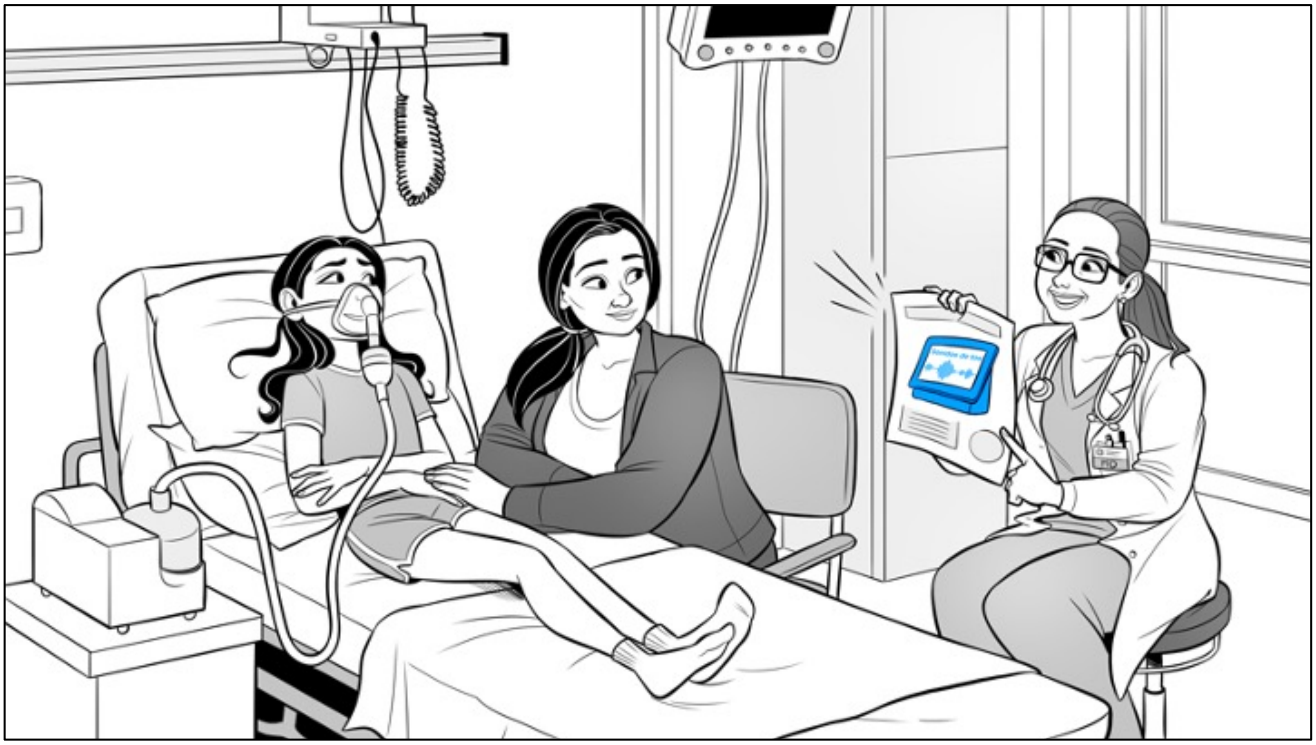

El facilitador del grupo focal lee en voz alta:
Entonces, María llevó a Sofía a la sala de emergencias. Después de recibir tratamiento para su ataque de asma, Sofía estaba respirando bien de nuevo. La médica le recomendó a María que considerara obtener un monitor de tos para Sofía. María decide conseguir uno.

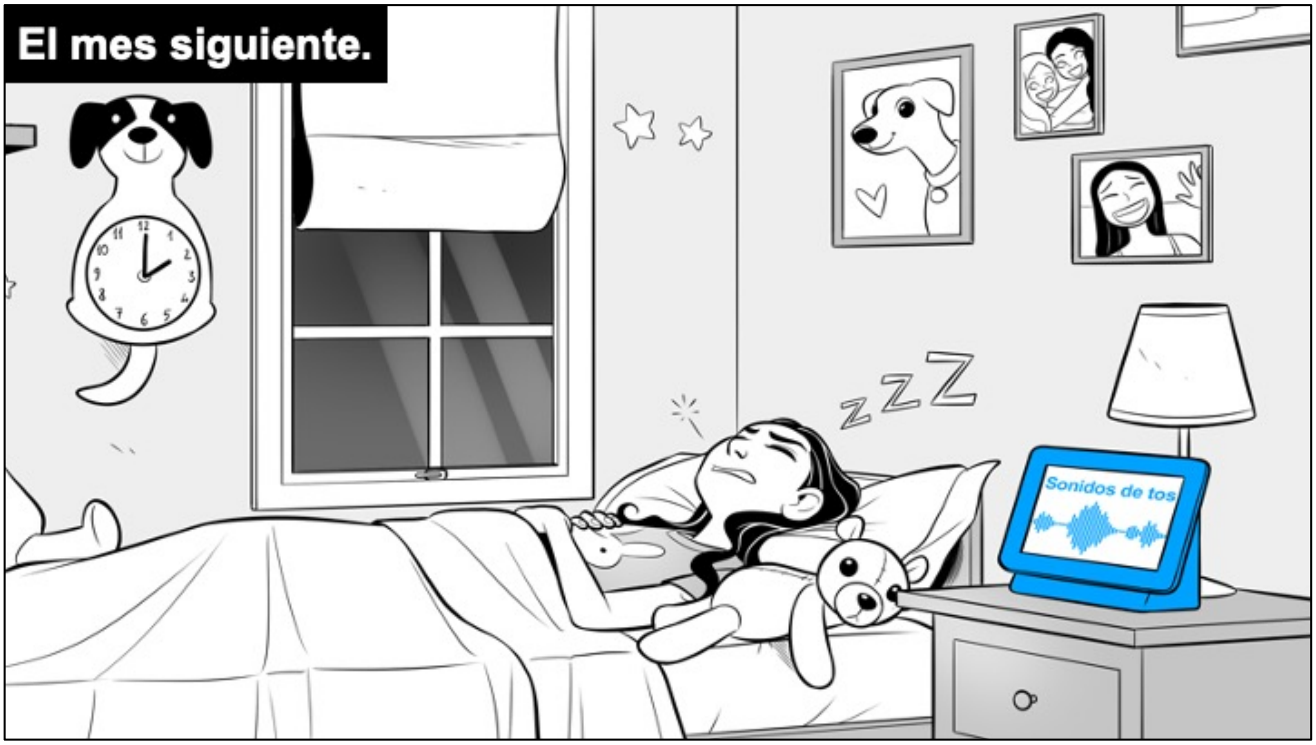

El facilitador del grupo focal lee en voz alta: El mes siguiente, María instala el monitor de tos en el dormitorio de Sofía. Este monitor captura el sonido durante toda la noche, registrando y revisando cualquier sonido de tos. Si registra mucha tos, envía una alerta que predice que Sofía podría estar a punto de tener un ataque de asma.

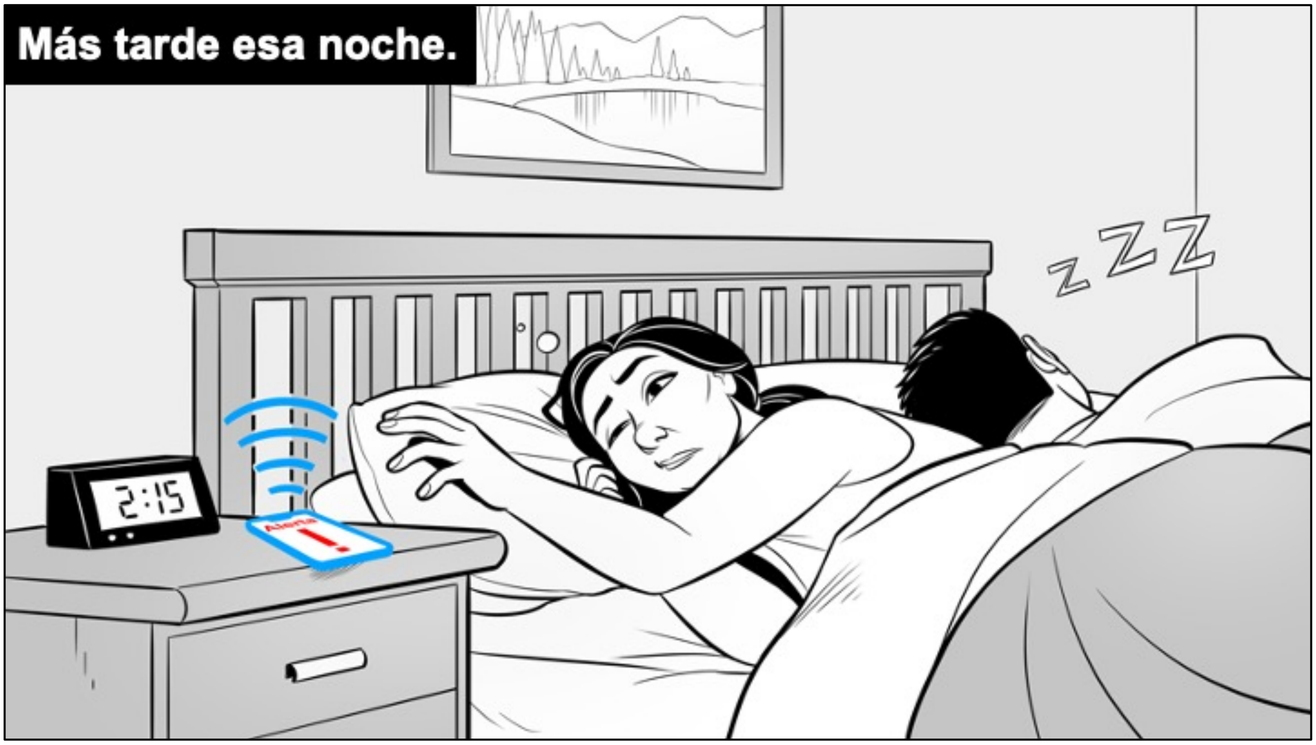

El facilitador del grupo focal lee en voz alta: Más tarde esa noche, el monitor de tos detecta que Sofía había comenzado a toser mucho, por lo que envía una alerta al teléfono de María. Esto la despierta para que pueda ayudar a Sofía.

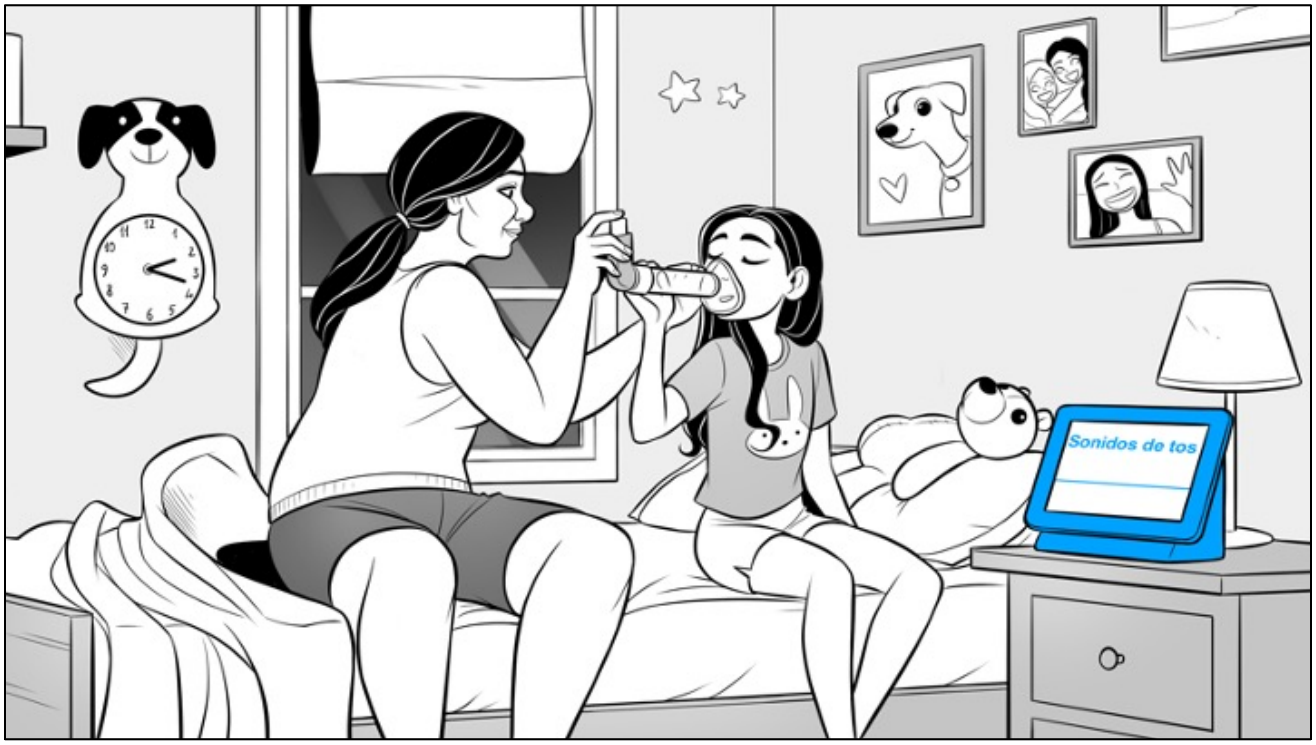

El facilitador del grupo focal lee en voz alta:
María ayuda a Sofía a usar su inhalador rápidamente. Después de tomar el medicamento, Sofía deja de toser y no tienen que ir a la sala de emergencias.

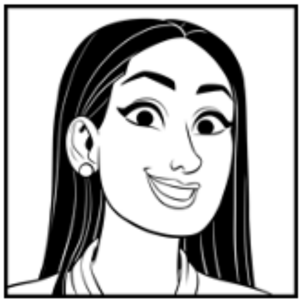

Diana

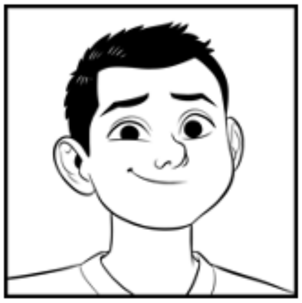

Arturo

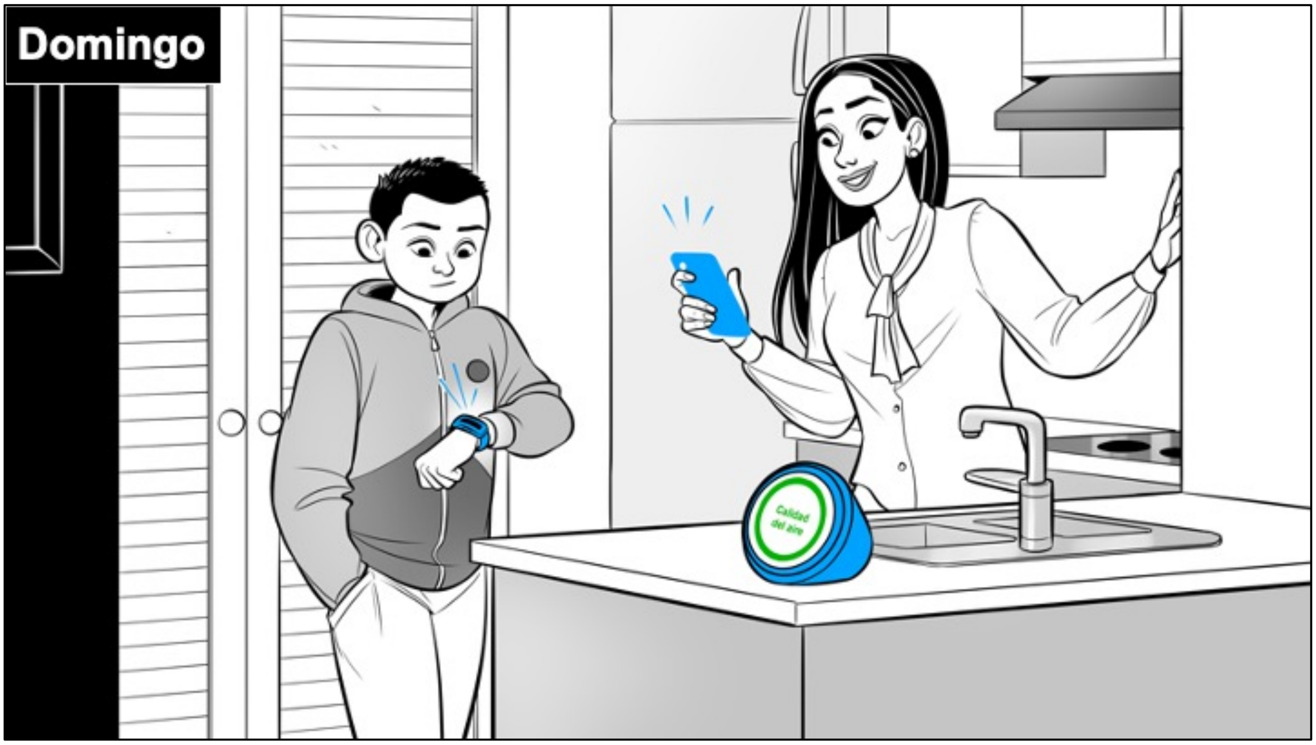

El facilitador del grupo focal lee en voz alta:
Ellos son Diana y su hijo Arturo. Arturo tiene 13 años y tiene asma. A Arturo le encanta el fútbol y su equipo jugará el campeonato estatal la próxima semana. Arturo quiere practicar todo lo que pueda antes del gran partido. La mayor preocupación de Diana es mantener el asma de Arturo bajo control.

Ella usa un poco de tecnología para hacer esto:

- un monitor de calidad del aire;
- un *smartwatch* con monitor de tos que Arturo

usa todos los días;
- y una aplicación en su teléfono donde puede rastrear cada vez que Arturo usa su inhalador.

Al igual que otras tecnologías, estos dispositivos tienen una variedad de precios, de más a menos costosos.

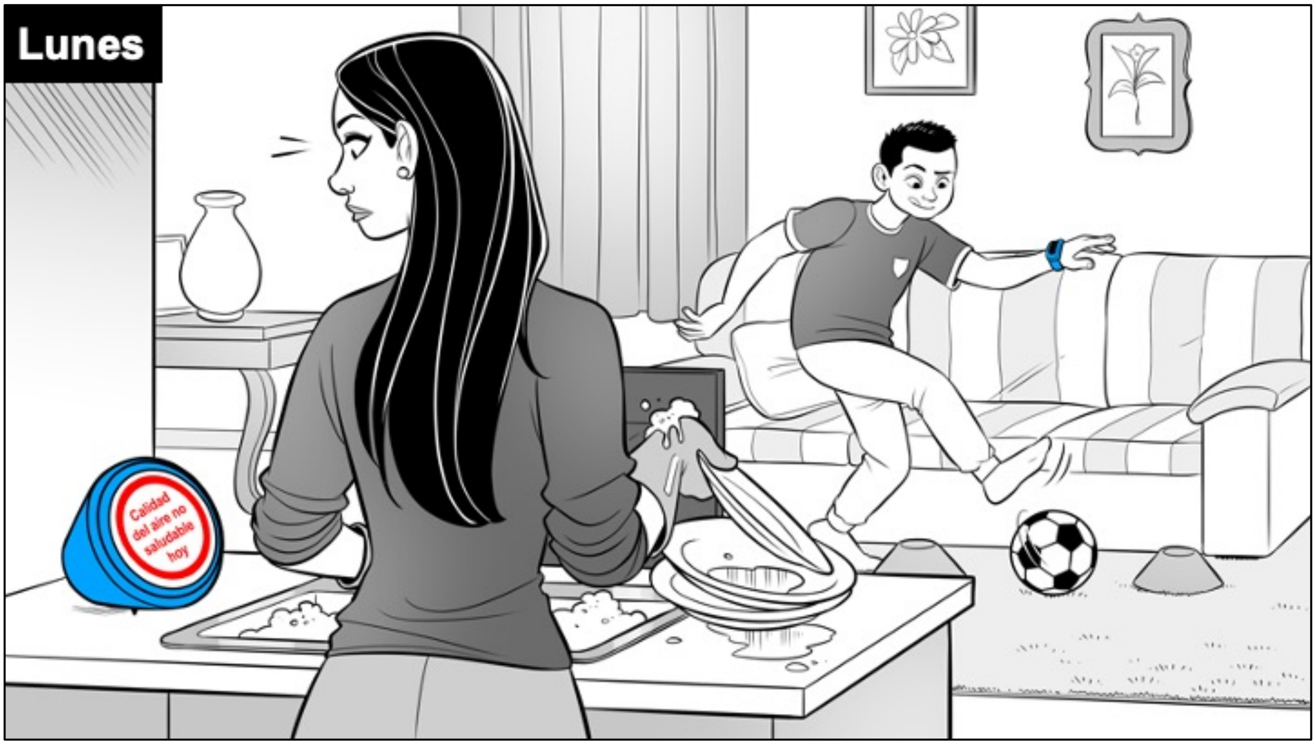

El facilitador del grupo focal lee en voz alta: El lunes, el monitor de calidad del aire muestra que el aire exterior no es saludable porque hay mucha contaminación en el aire. Diana sabe que esto es malo para el asma de Arturo, y le dice a Arturo que no haga sus ejercicios afuera. Él está de acuerdo y hace algunos ejercicios de fútbol adentro de la casa para estar listo para el gran juego.

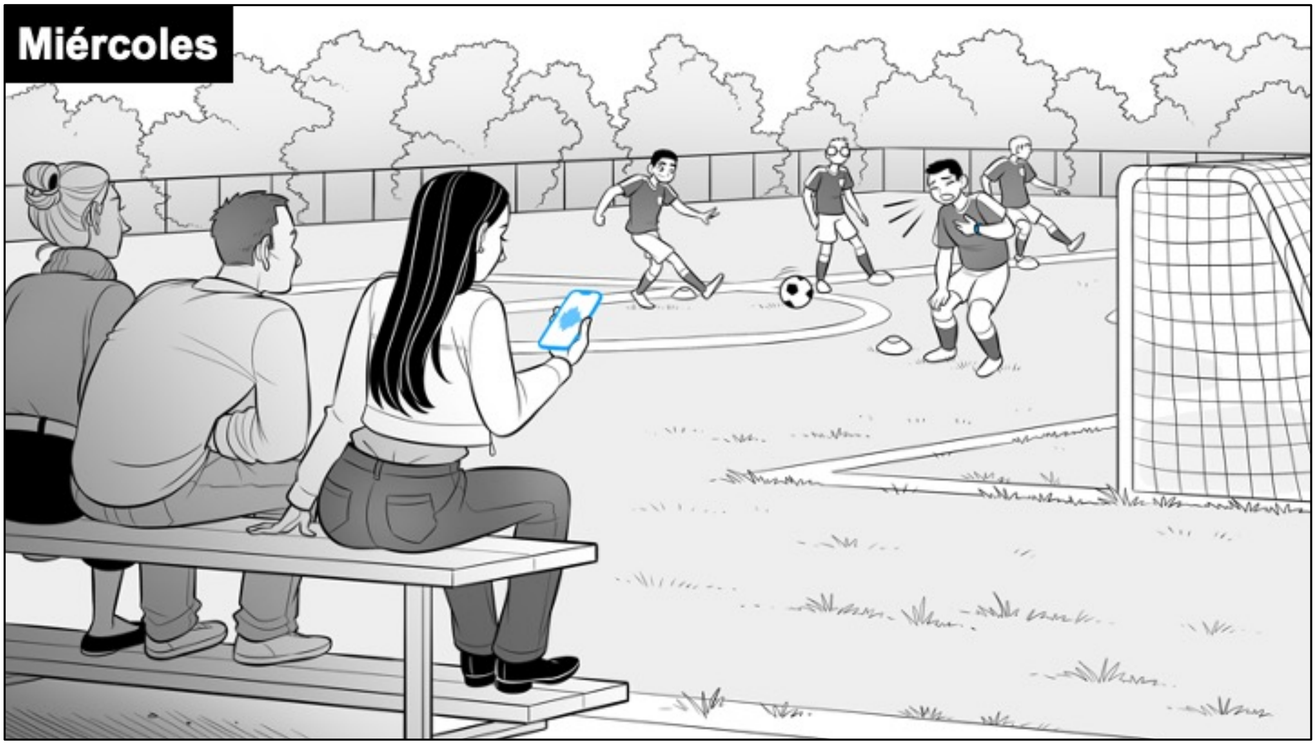

El facilitador del grupo focal lee en voz alta: El miércoles, Arturo va a la práctica de fútbol. Diana observa desde el margen. Ella revisa la aplicación del monitor de tos en su teléfono. La aplicación muestra que Arturo ha estado tosiendo más durante la noche de esta semana. Ella también nota que Arturo está tosiendo mucho durante la práctica de hoy, a pesar de que ya tomó su dosis normal antes del ejercicio de su inhalador de rescate. Diana le trae a Arturo su inhalador de rescate para una dosis adicional.

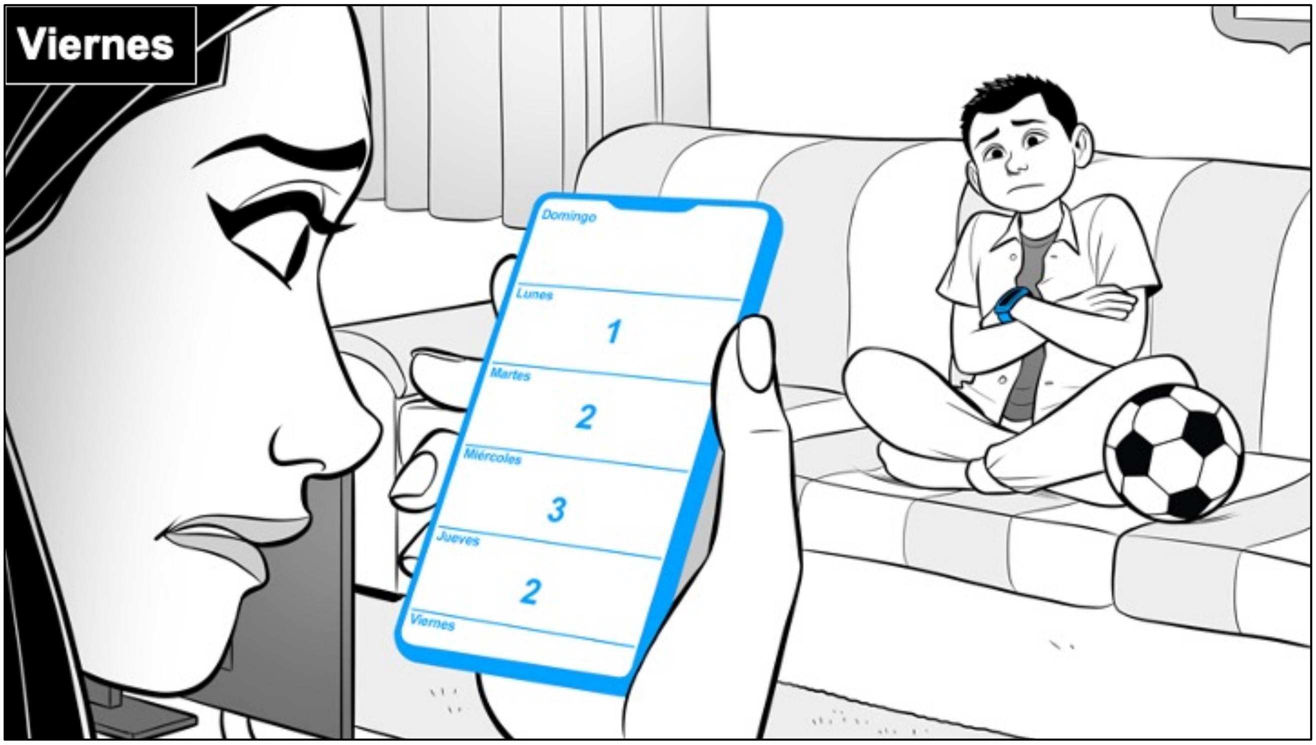

El facilitador del grupo focal lee en voz alta: El viernes por la noche, Diana mira la aplicación de monitoreo de su inhalador donde rastrea cada vez que Arturo usa su inhalador de rescate. Muestra que Arturo usó su inhalador 4 veces esta semana. También sabe que Arturo ha estado tosiendo más de lo normal esta semana, base al monitor de tos de su *smartwatch*. Quedan pocas prácticas antes del campeonato. Diana decide que Arturo no debe ir a las prácticas. En vez, Arturo se queda en casa y usa más su medicamento unos días para ayudar a contralar su asma.

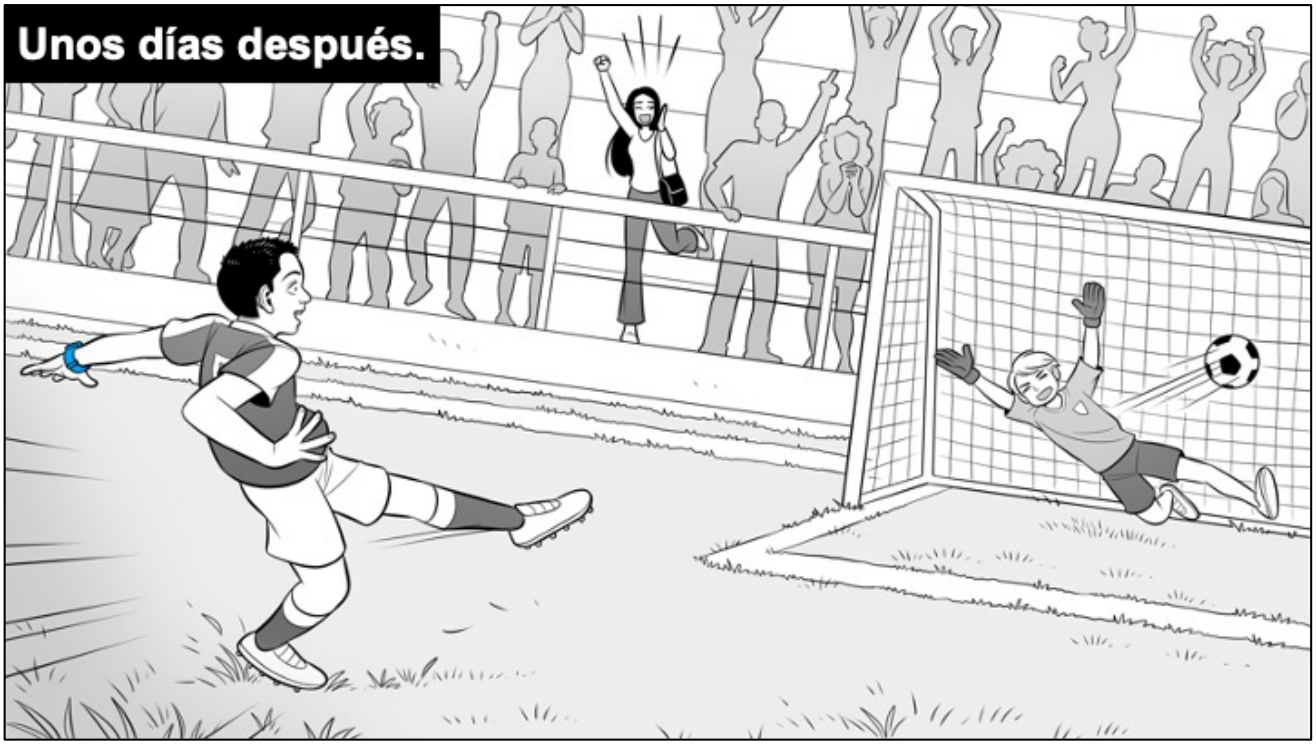

El facilitador del grupo focal lee en voz alta:
Después de que Arturo falto a las últimas prácticas y tomó su medicamento para controlar el asma, su respiración está mejor. Hoy, puede jugar en el campeonato estatal. Diana se siente bien por su decisión de mantenerlo fuera de la práctica. Ella siente que si no hubiera tomado esa decisión, Arturo no hubiera podido jugar hoy.

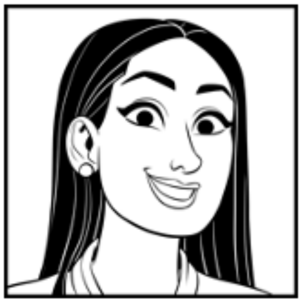

Diana

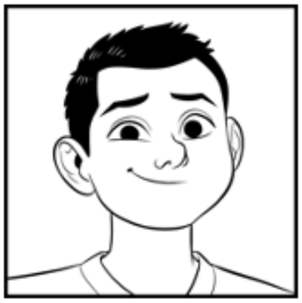

Arturo

Continuación

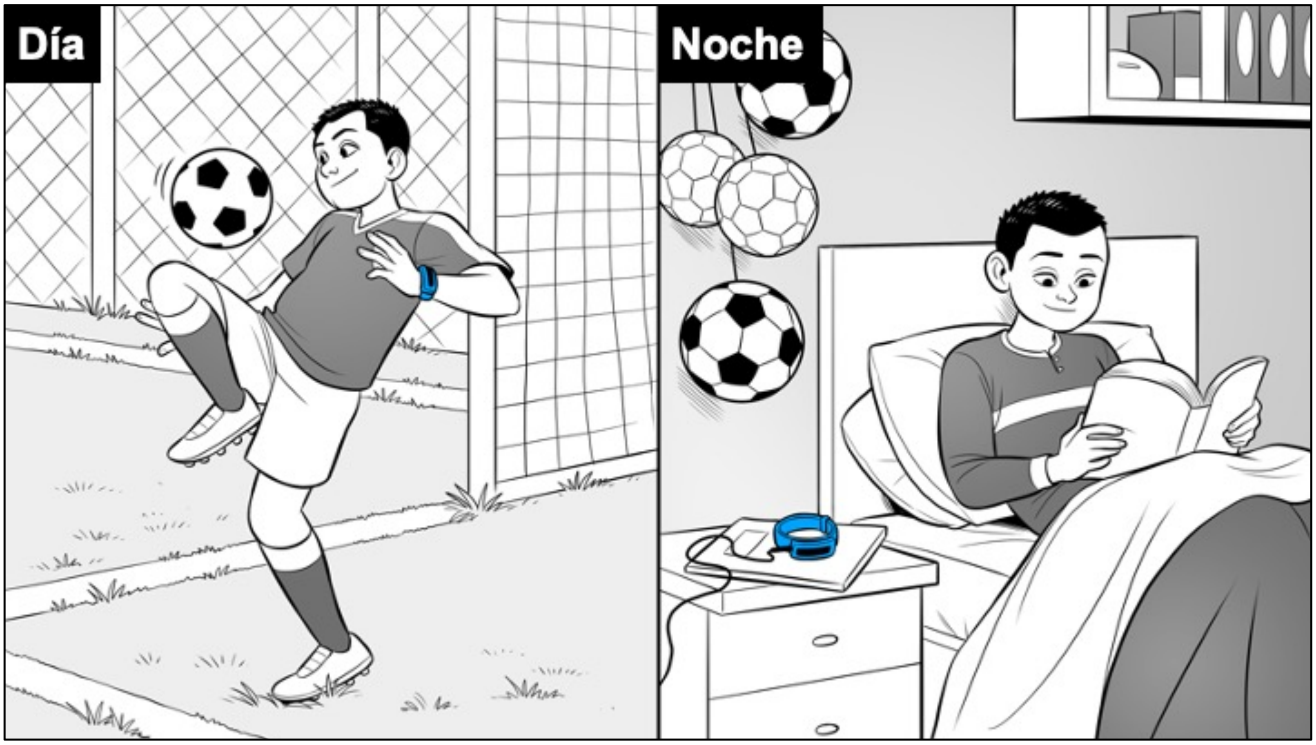

El facilitador del grupo focal lee en voz alta:
Cuando Arturo obtuvo por primera vez su smartwatch con monitor de tos, el reloj tuvo que aprender el ritmo normal de tos de Arturo. Usaba el reloj todo el día y lo ponía al lado de su cama por la noche. La aplicación aprendió el ritmo normal de tos de Arturo por el día y por la noche. Esta información ayudó al monitor de tos a saber cuándo Arturo está tosiendo más de lo normal. Esto le permitió aprender a predecir cuándo es más probable que Arturo tenga un ataque de asma.

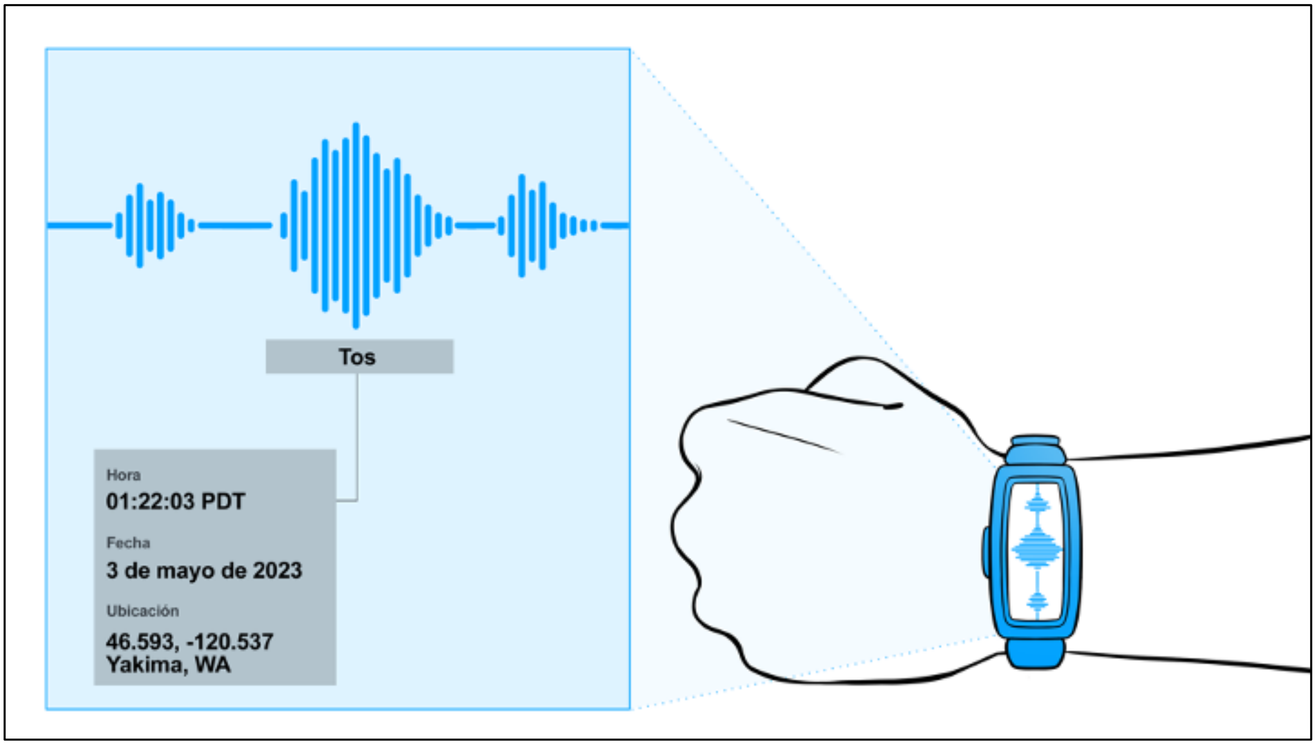

El facilitador del grupo focal lee en voz alta: El monitor analiza el sonido de la tos de Arturo y registra su ubicación. Los monitores de tos solo detectan la tos, no el habla u otro sonido. Cuando el monitor de tos identifica que Arturo ha estado tosiendo más de lo normal y puede estar en riesgo de un ataque de asma, lo alerta. Pero Arturo no es la única persona que obtiene información de su monitor de tos.

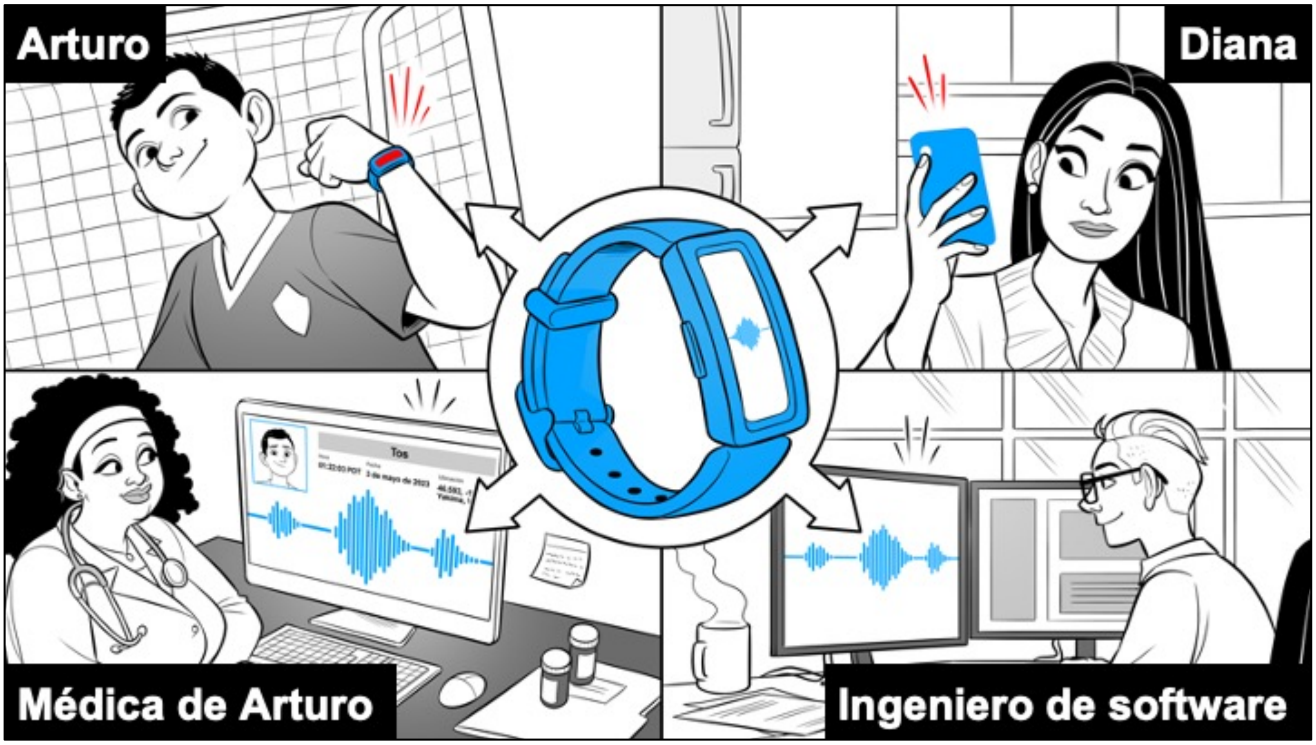

El facilitador del grupo focal lee en voz alta:
Aquí están todas las personas que reciben alertas e información del monitor de tos de Arturo.

- Arturo recibe alertas en su reloj.
- Diana recibe alertas e información en su teléfono.
- La médica de Arturo también puede obtener información del monitor de tos. Diana configuró la aplicación para enviar a la médica la información que colecta el monitor de tos, como

la hora de la tos y la ubicación de Arturo.

- La compañía que fabrica la aplicación para controlar la tos también obtiene información. Obtienen información de todas las personas que usan su aplicación. Esta información les ayuda a mejorar su aplicación. Por ejemplo, a veces los monitores de tos pueden tener una falsa alarma: predicen un alto riesgo de un ataque de asma incluso cuando el asma de la persona está bajo control. Usando la información de las personas que tienen su aplicación, la compañía puede actualizar la aplicación para que pueda predecir mejor el riesgo de ataques de asma.

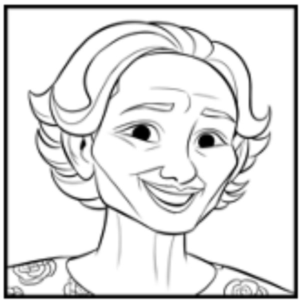

Dolores

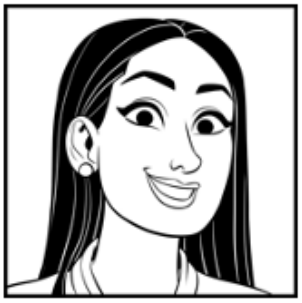

Diana

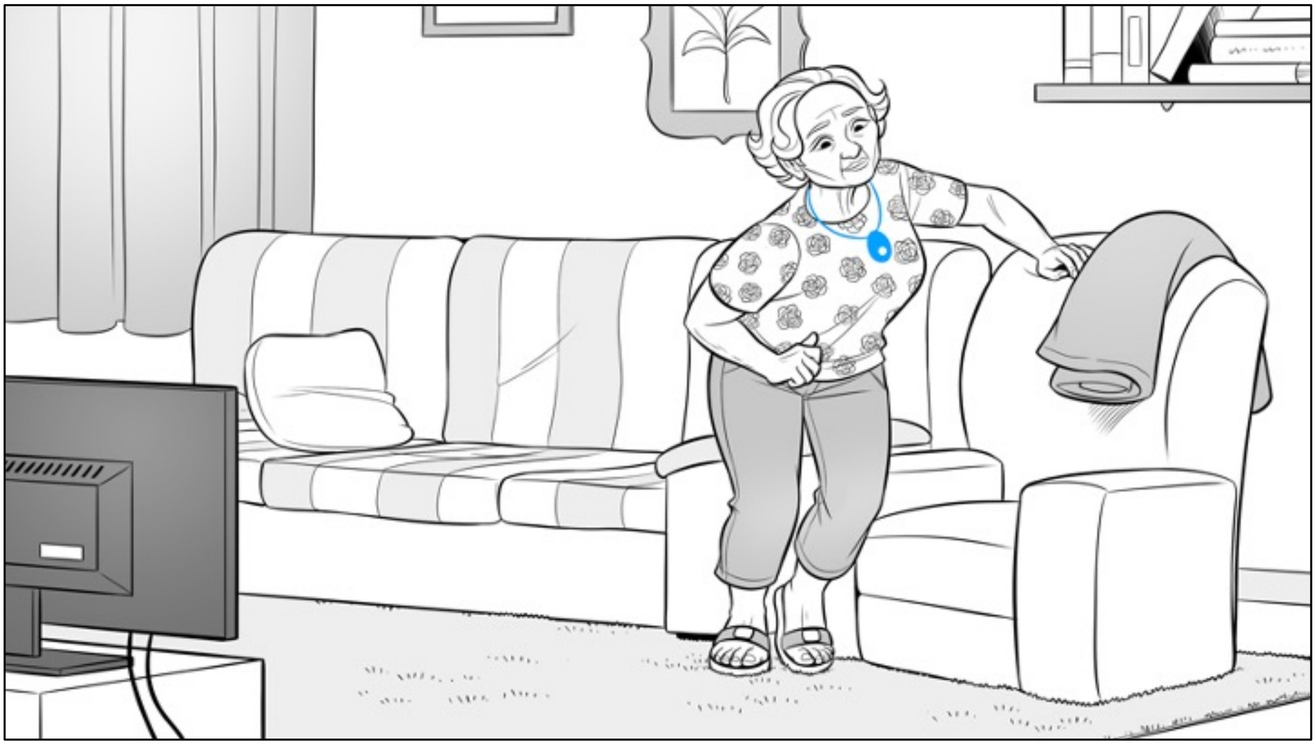

El facilitador del grupo focal lee en voz alta: Esta es la abuelita de Diana, Dolores. Dolores tiene casi 90 años y vive con la familia de Diana. Diana siempre está preocupada de que Dolores pueda caerse porque su equilibrio ha ido empeorando a medida que envejece. Ahora que Diana ha tomado un turno extra en el trabajo, Dolores tiene que estar sola en casa parte del día. Por eso, Diana compró un dispositivo de rastreo que Dolores puede usar como un collar. El dispositivo aprende los patrones de movimiento típicos, a predecir cuándo está en riesgo de caerse. Si el dispositivo detecta que Dolores tiene problemas con el equilibrio, puede enviar alertas a Diana y a la hermana de Diana. Si

el dispositivo detecta que Dolores se cae, también puede enviar alertas a los servicios de emergencia sin que nadie tenga que marcar el 911.
